# Rehabilitation of motor function after stroke: A bibliometric analysis of global research from 2004 to 2022

**DOI:** 10.3389/fnagi.2022.1024163

**Published:** 2022-11-02

**Authors:** Jinjing Hu, Jihua Zou, Yantong Wan, Qiuru Yao, Peng Dong, Gege Li, Xuan Wu, Lijie Zhang, Donghui Liang, Qing Zeng, Guozhi Huang

**Affiliations:** ^1^Department of Rehabilitation Medicine, Zhujiang Hospital, Southern Medical University, Guangzhou, China; ^2^School of Rehabilitation Medicine, Southern Medical University, Guangzhou, China; ^3^College of Anesthesiology, Southern Medical University, Guangzhou, China; ^4^School of Nursing, Southern Medical University, Guangzhou, China; ^5^Department of Traditional Chinese Medicine, Zhujiang Hospital, Southern Medical University, Guangzhou, China

**Keywords:** CiteSpace, VOSviewer, stroke, motor function, rehabilitation

## Abstract

**Background and aims:**

The mortality rate of stroke has been increasing worldwide. Poststroke somatic dysfunctions are common. Motor function rehabilitation of patients with such somatic dysfunctions enhances the quality of life and has long been the primary practice to achieve functional recovery. In this regard, we aimed to delineate the new trends and frontiers in stroke motor function rehabilitation literature published from 2004 to 2022 using a bibliometric software.

**Methods:**

All documents related to stroke rehabilitation and published from 2004 to 2022 were retrieved from the Web of Science Core Collection. Publication output, research categories, countries/institutions, authors/cocited authors, journals/cocited journals, cocited references, and keywords were assessed using VOSviewer v.1.6.15.0 and CiteSpace version 5.8. The cocitation map was plotted according to the analysis results to intuitively observe the research hotspots.

**Results:**

Overall, 3,302 articles were retrieved from 78 countries or regions and 564 institutions. Over time, the publication outputs increased annually. In terms of national contribution, the United States published the most papers, followed by China, Japan, South Korea, and Canada. Yeungnam University had the most articles among all institutions, followed by Emory University, Fudan University, and National Taiwan University. Jang Sung Ho and Wolf S.L. were the most productive (56 published articles) and influential (cited 1,121 times) authors, respectively. “Effect of constraint-induced movement therapy on upper extremity function 3–9 months after stroke: the Extremity Constraint Induced Therapy Evaluation randomized clinical trial” was the most frequently cited reference. Analysis of keywords showed that upper limbs, Fugl–Meyer assessment, electromyography, virtual reality, telerehabilitation, exoskeleton, and brain–computer interface were the research development trends and focus areas for this topic.

**Conclusion:**

Publications regarding motor function rehabilitation following stroke are likely to continuously increase. Research on virtual reality, telemedicine, electroacupuncture, the brain–computer interface, and rehabilitation robots has attracted increasing attention, with these topics becoming the hotspots of present research and the trends of future research.

## Introduction

Stroke is a cerebrovascular disease typically caused by the rupture or obstruction of blood vessels in the brain. The interruption of blood flow to the brain induces brain tissue damage (Isaacs-Itua and Wong, 2021). The global incidence risk of stroke has been increasing, and stroke has become a major cause of death (Stinear et al., 2020). Motor dysfunction is a common sequela in patients after recovery from stroke, accounting for ~50%–70% of all sequelae (Borges et al., 2018). Motor function is the primary evaluation index and the most critical problem in responding to the complaints of such patients. Improving the motor function of patients is necessary to enhance the quality of life, reduce social effects and socioeconomic expenses, and decrease the incidence of disability. Rehabilitation therapy is frequently used to rehabilitate motor function after stroke (Le Danseur, 2020). Effective rehabilitation strategies can manipulate neuroplasticity to strengthen the motor function of the patients and improve their quality of life (Dimyan and Cohen, 2011). Recently, various treatment methods, including sports, music, and mirror therapies, have gradually been introduced in the rehabilitation of motor function after stroke (Daniel et al., 2021; Saavedra-García et al., 2021; Timmermans et al., 2021). More scientists are focusing on rehabilitating motor function after stroke and have published several articles regarding the same; however, the direction and hotspots of research in this field remain unclear. It is necessary to conduct a bibliographic analysis of research on motor function rehabilitation after stroke to fully utilize the existing research foundation and identify the research hotspots.

The published bibliometric analyses report the temporal trends, geographic distribution, and socioeconomic determinants of scientific production related to cerebrovascular and cardiovascular disease rehabilitation (Ugolini et al., 2013). Furthermore, there exists a bibliometric analysis regarding the effectiveness of rehabilitation treatment and a quantitative analysis regarding exercise interventions for stroke (Feng et al., 2013; Dong et al., 2022) However, to the best of our knowledge, there has been no detailed bibliometric analysis regarding the rehabilitation of motor function after stroke, thereby necessitating qualitative and quantitative analyses on motor function rehabilitation after stroke. Literature metrology is a widely accepted statistical method. It considers the writing framework and estimation qualities the exploration article and uses numerical, factual, and other estimation techniques to depict the collected data and patterns in particular research fields (Osareh, 1996). Recently, bibliometrics have provided clear insights into several biological areas, such as ophthalmic, health information and environmental science fields (Efron, 2021; Kokol et al., 2021; Zhou et al., 2021). However, from a bibliometric perspective, the comprehensive knowledge structure, evolutionary pathways, and research hotspots of motor function rehabilitation after stroke have not been analyzed. Bibliometric research on this topic will complement the existing knowledge regarding motor function rehabilitation after stroke and intuitively provide researchers with visual information and potential research directions, promoting the further development of motor function rehabilitation technologies. Hence, this study performed a statistical analysis of articles related to stroke motor function rehabilitation published in the past 18 years and introduced the basic situation and development trends of motor rehabilitation after stroke, providing direction for further research.

## Materials and methods

### Data collection

Science Citation Index Expanded of the Web of Science Core Collection (WoSCC) was selected as the source database for data retrieval. WoSCC is an online database contains the standardized and latest reference datasets for scientific study and analysis, with SCIEXPANDED considered the most suitable data bank for bibliometric analysis (Chen et al., 2020; Yao et al., 2020).

**Figure 1:**
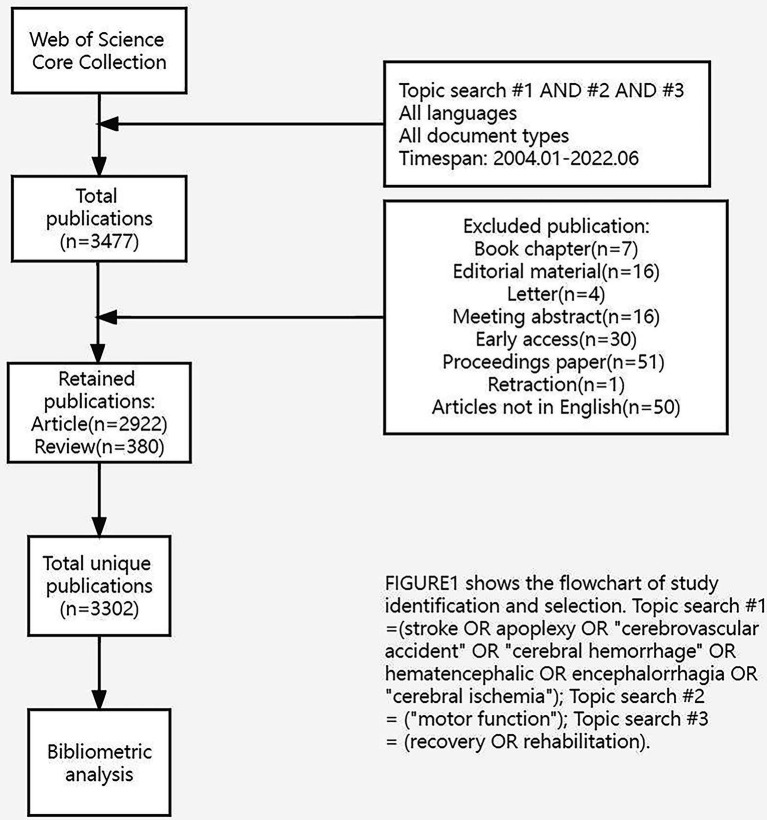
The flowchart of study identification and selection.

The following were the data retrieval strategies: TS = (stroke OR apoplexy OR “cerebrovascular accident” OR “cerebral hemorrhage” OR hematencephalic OR encephalorrhagia OR “cerebral ischemia”) AND TS = (“motor function”) AND TS = (recovery OR rehabilitation). To eliminate deviations, articles or reviews published from 2004 to 2022 were systematically searched between January 1, 2004 and June 23, 2022, and downloaded in 1 day (June 23, 2022). Some irrelevant literature was excluded, including proceedings papers, early access, and book chapters. In total, 3,302 original English articles (including 2,922 articles and 380 reviews) were screened. The flowchart in Figure 1 illustrates the identification and selection processes of the studies.

The complete records of each article, including publication outputs, research categories, countries/institutions, authors/cocited authors, journals/cocited journals, cocited references, and keywords, were downloaded from the WoSCC database in text format.

### Data analysis

We combined the same author names and synonymous keywords and removed any spelling errors. Subsequently, the processed data were imported to CiteSpace version 5.8 and VOSviewer v.1.6.15.0 for bibliometric analysis.

CiteSpace visualizes and analyzes citations and provides an intuitive understanding of the research hotspots and evolution of various areas of a particular field and predicts its future development (Synnestvedt et al., 2005). Instead of focusing on the partial contributions of specific papers, close attention was paid to their academic influence on the overall development of the field. CiteSpace dissected the essential data of the articles, including countries, institutions, cocited references, and reference burst, by producing visual maps. In addition, cocitations and coreferences were visualized to analyze collaborations among the recognized distributions (Dong et al., 2022).

VOSviewer constructs and visualizes bibliometric maps. VOSviewer was primarily used to analyze the bibliometric network, construct a visual organization map, and understand the structural and dynamic development of academic research (Ma et al., 2021). VOSviewer was also used to visually analyze the authors, research categories, countries, journals, keywords, and timelines and identify the frontier hotspots.

## Results

### Publication trends

**Figure 2:**
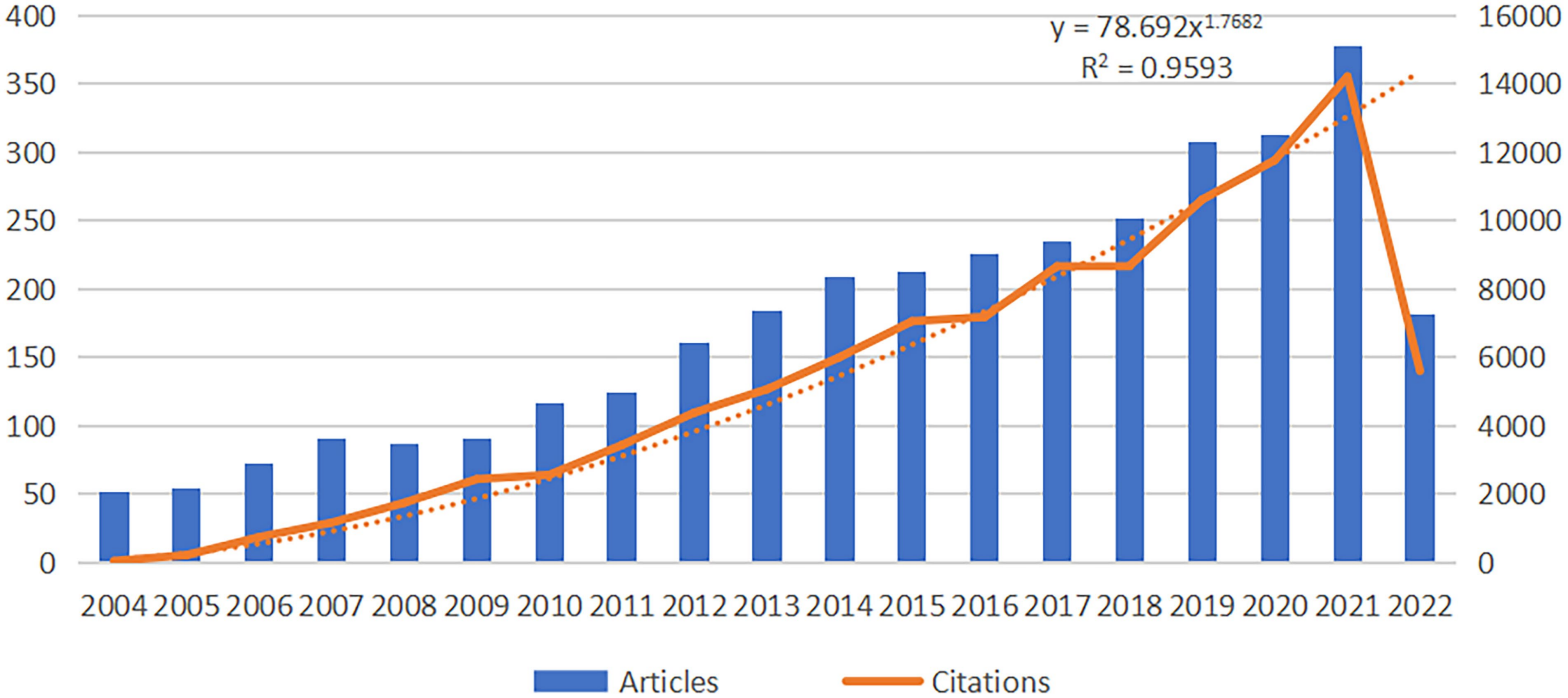
Annual publication trend of stroke motor function rehabilitation. The data for 2022 is not complete.

The distribution of articles in every period indicates the general examination patterns in the field. Figure 2 shows that the number of articles on motor function rehabilitation after stroke exhibits an increasing trend yearly. From 2004 to 2009, the number of publication outputs gradually increased. From 2009 to 2021, the number of documents steadily and rapidly increased, indicating that motor rehabilitation after stroke had received particular attention in that timeframe. In 2021, the publication output was 377. Although complete information for 2022 is not yet available, it is estimated that the final data for 2022 will show a rapid growth trend compared to before.

### Country/region and institution contributions

**Figure 3:**
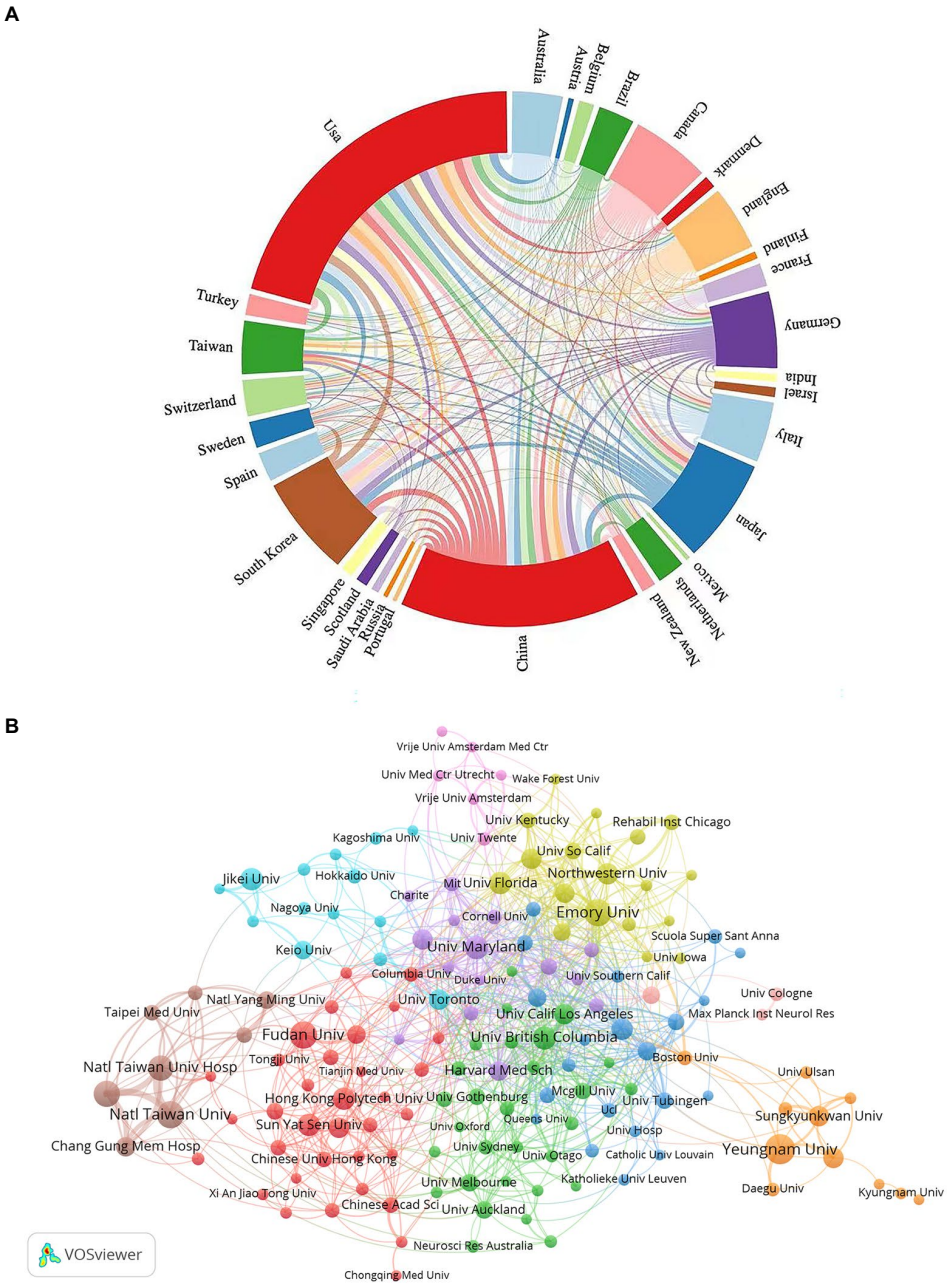
**(A)** countries/regions clustering analysis; **(B)** institution’s clustering analysis.

Overall, 3,302 articles from 78 countries or regions and 564 institutions had been published. As depicted in Table 1, the United States contributed the most papers in this field (953 publications, 28.86%), followed by China (674 publications, 20.41%), implying that the contributions of US and China were significantly more than that of the other countries in this field, followed by Japan (293 publications, 8.87%), South Korea (282 publications, 8.54%), and Canada (222 publications, 6.72%). We used CiteSpace for the visual analysis of the countries or regions (Supplementary Figure S1A). The size of the circle denotes the number of articles distributed by countries or regions, and the line reflects the strength of cooperation between the countries or regions. The purple circle outside the circle represents intermediate centrality. Additionally, cluster analysis was performed for countries or regions (Figure 3A). The size of the circle was directly proportional to the number of articles published in that country/region. The United States had the highest centrality, followed by England, Germany, Canada, Australia, France, Belgium, and Spain. The higher the centrality of a node, the more times it appears in the shortest path of the whole network and greater its influence and significance.

**Table 1:** Top 10 countries/regions and institutions related to stroke motor function rehabilitation.

Rank	Country/Region	Total link strength	Count (%)	Institution	Total link strength	Count (%)
1	United States	527	953 (28.86%)	Yeungnam Univ	32	76 (2.30%)
2	China	204	674 (20.41%)	Emory Univ (United States)	106	66 (1.99%)
3	Japan	188	293 (8.87%)	Fudan Univ (China)	36	63 (1.91%)
4	South Korea	163	282 (8.54%)	Natl Taiwan Univ (China)	139	63 (1.91%)
5	Canada	160	222 (6.72%)	Chang Gung Univ (China)	119	59 (1.79%)
6	Germany	157	214 (6.48%)	Natl Taiwan Univ Hosp (China)	132	56 (1.69%)
7	England	118	181 (5.48%)	Univ British Columbia (Canada)	61	49 (1.48%)
8	Italy	116	169 (5.11%)	Univ Maryland (United States)	67	47 (1.42%)
9	Taiwan	93	149 (4.51%)	Hong Kong Polytech Univ (China)	31	44 (1.33%)
10	Australia	88	141 (4.27%)	Sun Yat Sen Univ (China)	28	43 (1.30%)

The institutions with the most number of articles are Yeungnam University (76 publications, 2.30%), followed by Emory University (66 publications, 1.99%), Fudan University (63 publications, 1.91%), and National Taiwan University (63 publications, 1.91%). In Supplementary Figure S1B, the size of the circle denotes the number of published articles by an institution. The purple circle represents centrality. Emory University and Harvard University showed high centrality. CiteSpace was also used to visually cluster analyze the institutions and those using keywords were marked. Various tones address various groups. As shown in Supplementary Figure S1C, the largest cluster is #0, labeled “CI therapy,” indicating that this term was used the most by the various institutions, followed by “electroacupuncture” (cluster #1), “telemedicine” (cluster #2), and “rehabilitation robotics” (cluster #3). Other significant groups included “neurorecovery,” “diffusion tensor imaging,” and “psychometrics.” The cluster analysis of the institutions is presented in Figure 3B. The hub size denotes the number of distributions of institutions, and the line represents the cooperation among the institutions. Node colors represent different clusters.

### Authors and cocited authors

**Figure 4:**
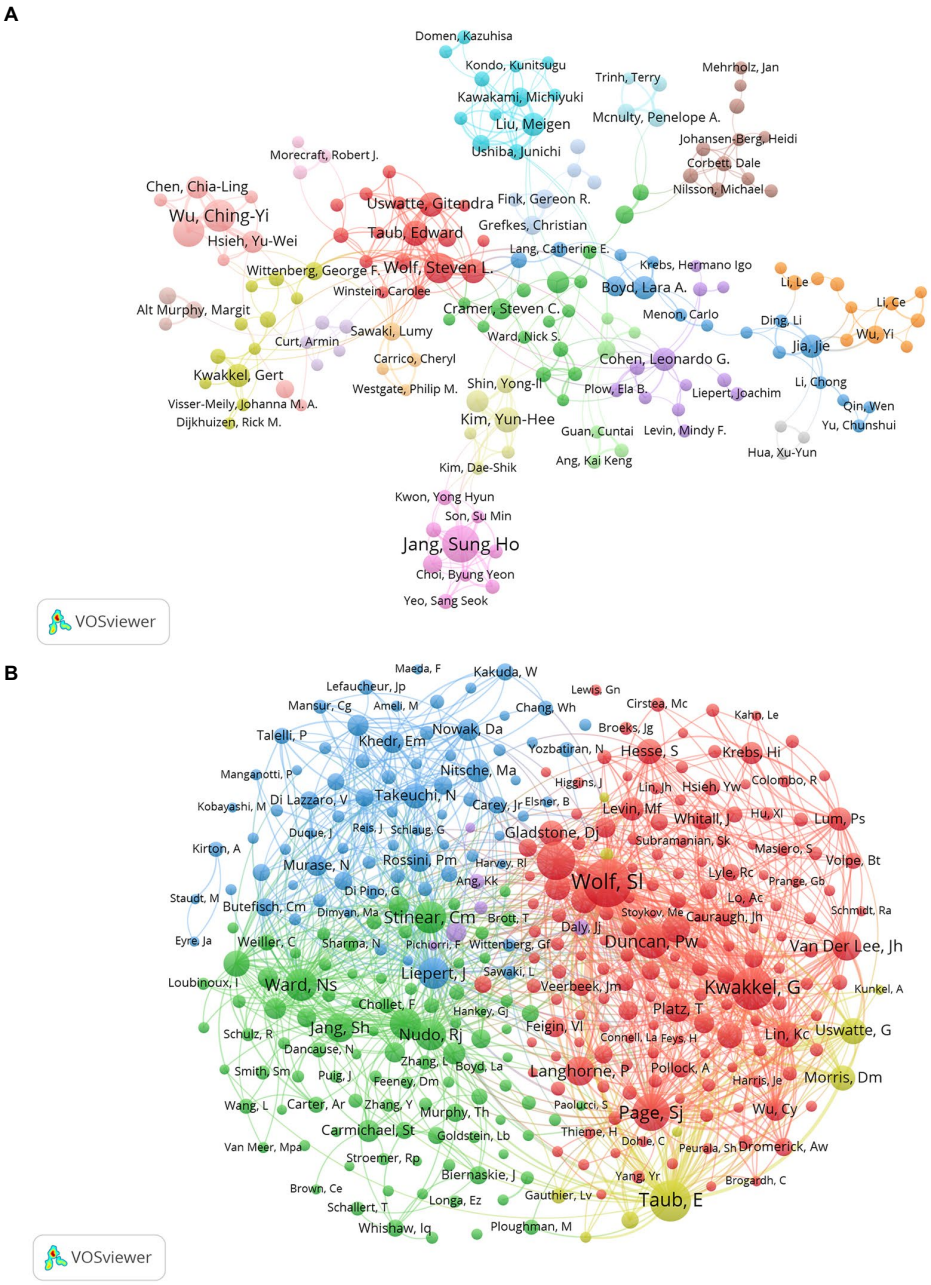
**(A)** Co-author related to stroke motor function rehabilitation; **(B)** co-cited author related to stroke motor function rehabilitation.

Altogether, 14,523 authors were associated with publications related to motor function recovery after stroke. Analyzing the authors of the literature enabled us to determine the representative scholars and core research forces in this field. As shown in Table 2, Sung Ho Jang had the largest number of distributed articles (56, 1.69%), followed by Ching-Yi Wu (41, 1.24%) and Keh-Chung Lin (39, 1.18%). Among the top 10 authors, Ching-Yi Wu (81) and Keh-Chung Lin (80) showed high total link strength, which indicated a close cooperative relationship of these authors with other authors. As shown in Figures 4A,B, each circle represents a different author, lines connecting the circles reflect connections between the authors, and variously colored networks of the linkages represent the groups of authors who frequently collaborate. Cocited authors were defined as at least two authors who are referred to in at least one ensuing paper, and they are said to have a cocitation relationship. Seven of the top 10 cocited authors were cited >500 times (Table 2). S. Wolf (1,121) was the most cited author, followed by G. Kwakkel (866) and E. Taub (827). Moreover, as shown in Supplementary Figure S2, the time of publication can be clearly and intuitively observed to understand the research hotspots in real time.

**Table 2:** Top 10 authors and co-cited authors related to stroke motor function rehabilitation.

Rank	Author	Count (%)	Total link strength	Co-cited author	Citation	Total link strength
1	Jang Sung Ho	56 (1.69%)	63	Wolf Sl	1,121	23,158
2	Wu Ching-Yi	41 (1.24%)	81	Kwakkel G	866	18,728
3	Lin Keh-Chung	39 (1.18%)	80	Taub E	827	17,892
4	Wolf Steven L.	37 (1.12%)	73	Fuglmeyer Ar	694	11,946
5	Kim Yun-Hee	25 (0.75%)	53	Page Sj	657	16,488
6	Taub Edward	25 (0.75%)	73	Duncan Pw	556	10,509
7	Winstein Carolee J.	24 (0.72%)	43	Ward Ns	504	12,209
8	Uswatte Gitendra	23 (0.69%)	72	Stinear Cm	496	11,750
9	Boyd Lara A.	22 (0.66%)	24	Liepert J	469	12,636
10	Cohen Leonardo G.	22 (0.66%)	30	Nudo Rj	462	10,270

### Research categories

**Figure 5:**
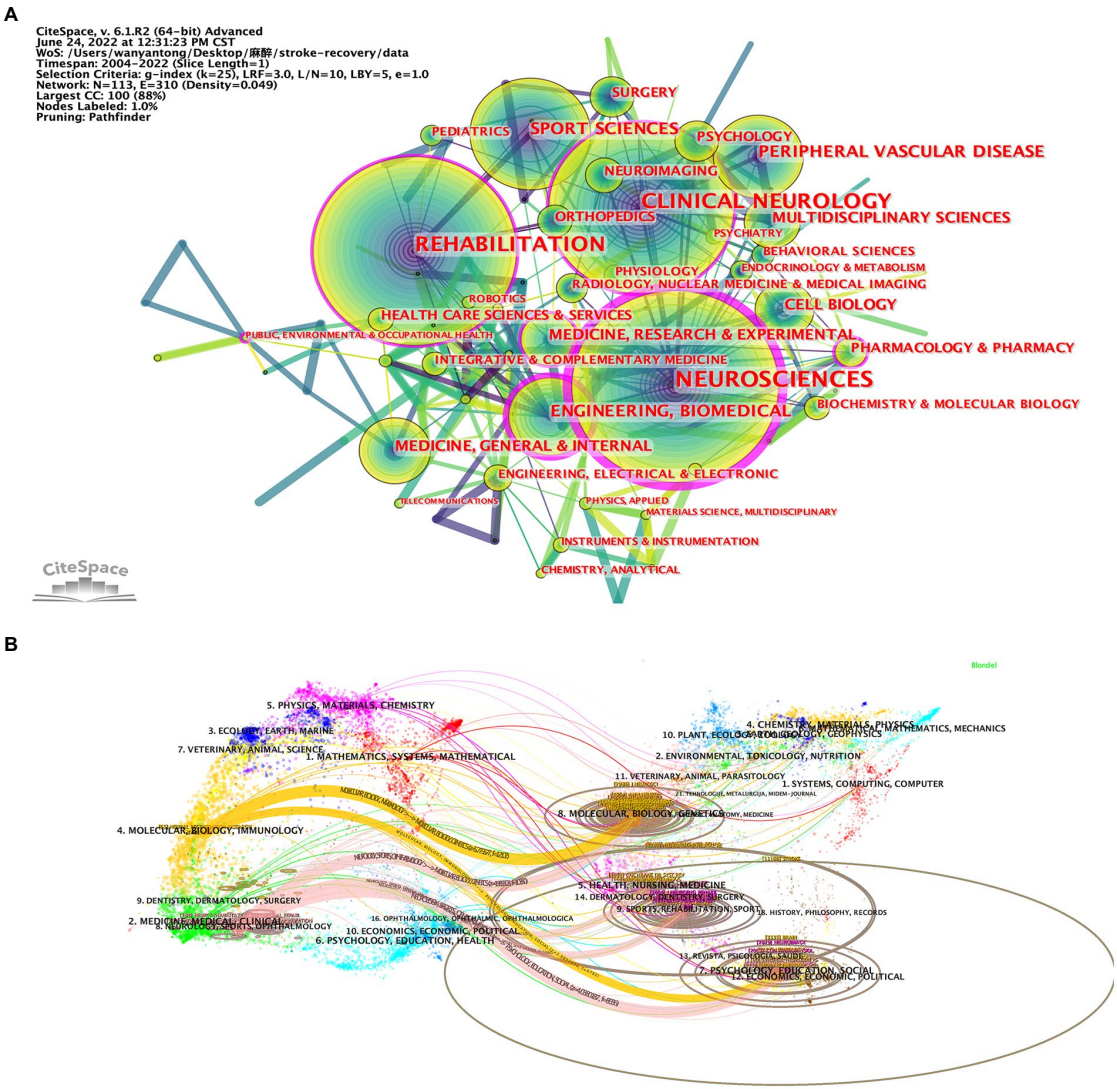
Category network map in the field of stroke motor function rehabilitation: **(A)** publication distribution among different categories; **(B)** dual-map overlay of journal publishing research.

The category graph generated using CiteSpace showed 113 nodes, suggesting that the field of study involves 113 categories. Moreover, the hub size represents the recurrence of the event, the lines between them denote the strength of the association, and the purple circle represents centrality (Figure 5A). The most frequently occurring and extensive classification was neuroscience, with a recurrence of 400, followed by rehabilitation (315 distributions). Other frequent categories include clinical neurology (231 distributions), sports sciences (195 distributions), and science and technology (193 distributions). Moreover, in the overlay of the dual map of research publishing in journals (Figure 5B), the left half of the diagram depicts the referring to diaries, the right half depicts the referred to diaries, and the bends constitute the reference lines that ultimately depict the reference setting. More articles a diary distributes, more extended the upward pivot of the oval, and the more writers, the more extended the event hub of the circle. We found six predominantly cited pathways (color orange, pink), showing that studies published in molecular, biological and genetic journals and in psychology, education and social science journals were predominantly cited by studies published in molecular, biological and immunological journals.

### Journals and coreferred scholarly periodicals

**Figure 6:**
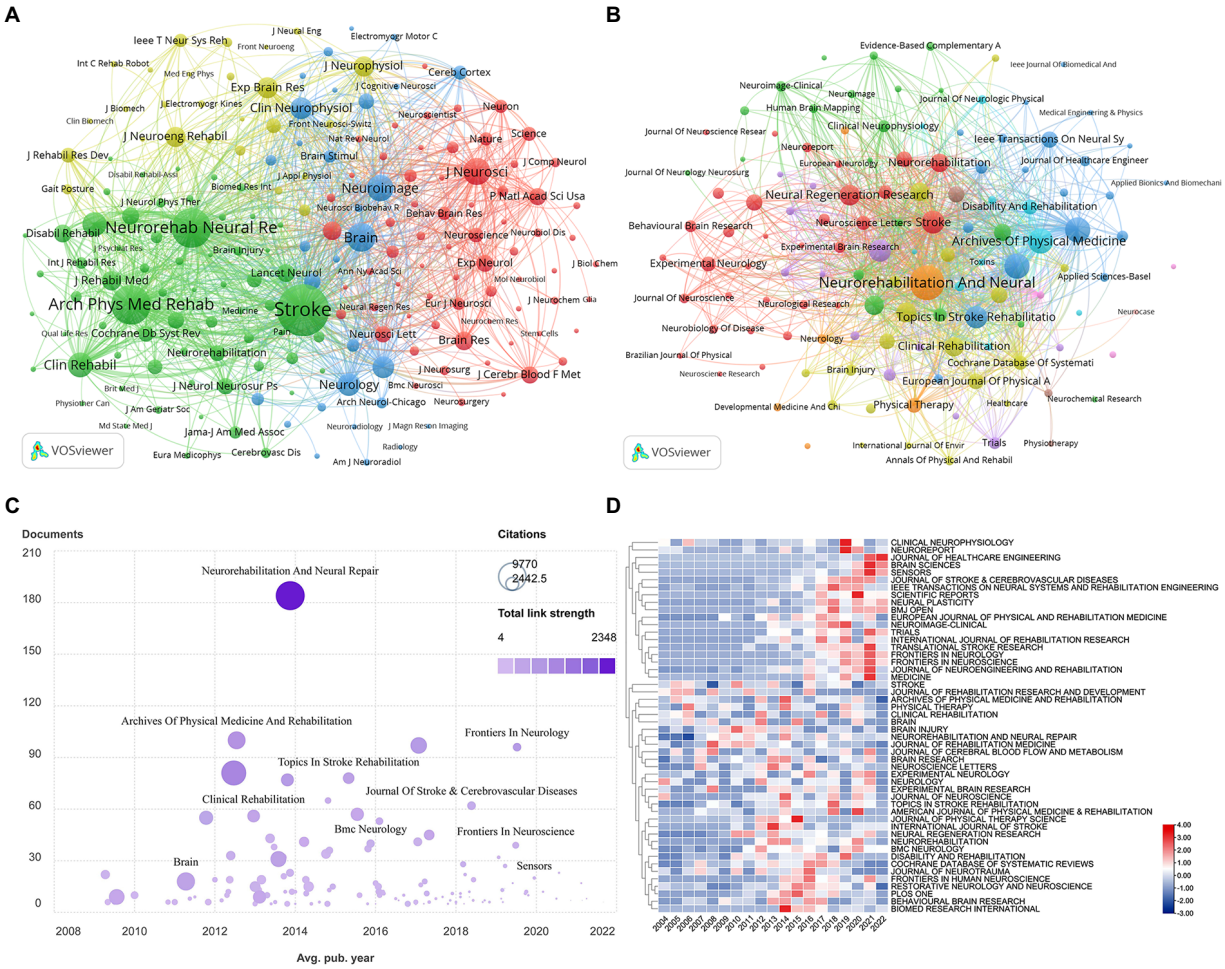
**(A)** Co-cited journals related to stroke motor function rehabilitation; **(B)** Journals related to stroke motor function rehabilitation; **(C)** number of journal publications, frequency of citations, total link strength over time; **(D)** journals heat map on stroke motor function recovery.

In total, 3,302 articles were distributed in 595 journals related to motor recovery after stroke. The distributed diaries were visually examined *via* VOSviewer programming. As presented in Table 3, the journal *Neurorehabilitation and Neural Repair* had the most number of articles (184, 5.57%), followed by *Archives of Physical Medicine and Rehabilitation* (100, 3.02%). Of the top 10 journals, *Stroke* (10.17) had the highest impact factor (IF). Of the top 10 coreferred to journals (Table 3), *Stroke* had the highest number of citations (11,443), indicating that the journal had important implications for motor function recovery after stroke, followed by *Archives of Physical Medicine and Rehabilitation* (6,427) and *Neurorehabilitation and Neural Repair* (6,321). The influence of journals depends on the number of joint citations. Additionally, more than half the journals belonged to Q1. As shown in Figures 6A,B, each circle represents a journal; the size of the circle depends on the strength of connection and number of references. The color of the circle denotes the different groups to which the journals belong, with the various bunches representing various categories. We can observe information, such as cooperation among journals, using this figure. The journals *Stroke*, *Archives of Physical Medicine and Rehabilitation*, and *Neurorehabilitation and Neural Repair* have more co-citations and greater influence. Figure 6C shows the number of journal publications, citation frequency, and overall strength of links over time, with larger circles indicating higher citation frequencies and darker colors indicating higher overall strength of links. *Neurorehabilitation and Neural Repair* has been cited frequently and has the highest total link strength.

**Table 3:** Top 10 journals and co-cited journals related to stroke motor function rehabilitation.

Rank	Journal	Count (%)	IF (2022)	JCR	Co-cited journal	Citation	IF (2022)	JCR
1	Neurorehabilitation and Neural Repair	184 (5.57%)	4.895	Q2	Stroke	11,170	10.17	Q1
2	Archives of Physical Medicine and Rehabilitation	100 (3.02%)	4.06	Q1	Arch Phys Med Rehab	6,427	4.06	Q1
3	Journal of Neuroengineering and Rehabilitation	97 (2.94%)	5.208	Q2	Neurorehab Neural Re	6,321	4.895	Q2
4	Frontiers in Neurology	96 (2.91%)	4.086	Q2	Brain	3,336	15.255	Q1
5	Stroke	81 (2.45%)	10.17	Q1	J Neurosci	2,986	6.709	Q1
6	Topics in Stroke Rehabilitation	78 (2.36%)	2.177	Q3	Neuroimage	2,836	7.4	Q1
7	Restorative Neurology and Neuroscience	77 (2.33%)	2.976	Q3	Clin Rehabil	2,634	2.884	Q2
8	Neural Regeneration Research	65 (1.96%)	6.058	Q2	Phys Ther	2,388	3.14	Q1
9	Journal of Stroke & Cerebrovascular Diseases	62 (1.88%)	2.677	Q4	Neurology	2,128	11.8	Q1
10	Plos One	57 (1.73%)	3.752	Q2	Clin Neurophysiol	2,006	4.861	Q2

Furthermore, we conducted a comparative analysis of the popularity of the journals (Figure 6D). Each line was compared with the one another. The value in the box denotes the number of articles distributed in the journal divided by the number of articles distributed per year, thereby representing the prevalence of the diary in the ongoing year. The popularity of the journals was illustrated using a heat map, wherein the intensity of the red color was used to indicate the popularity of a journal in a given year. Through this heat map, shifts in the exploration course, accentuation in this field, and developmental trends could be determined. For example, in 2014 and 2015, articles were mainly published in *Biome Research International* and *Journal of Physical Therapy Science*. From 2016 to 2020, several articles were published in *Clinical Neurophysiology*, *Neuroreport*, and *Scientific Reports*. From 2019 to 2022, *Medicine*, *Journal of Neuroengineering and Rehabilitation*, *Translational Stroke Research*, *Sensors*, *Brain Sciences*, and *Journal of Healthcare Engineering* had several publications. The direction of research in this field was observed to gradually shift from physical therapy to neurorehabilitation and neuroscience.

### Cocited references and reference burst

Of the 3,302 retrieved documents, the top 15 most frequently cited documents are presented in Table 4. “Effect of constraint-induced movement therapy on upper extremity function 3 to 9 months after stroke: the EXCITE randomized clinical trial” is the most frequently cited reference (1,303 citations). The main content of this paper is a prospective, single-blind, randomized, multisite clinical trial—EXCITE trial. The study reported that 2 weeks of CIMT improved upper-limb function in patients more than 1 year after stroke for at least a year. “Influence of interhemispheric interactions on motor function in chronic stroke” is the second most frequently cited reference (1,014 citations). This paper explored the interaction between the hemispheres during the autonomic development of deadened hands in patients with ongoing subcortical stroke and new corpus callosum. The third was Effects of robot-assisted therapy on upper limb recovery after stroke: A systematic review (948 citations), which was a systematic review of studies on the effects of robot-assisted therapy on motor and functional recovery in stroke patients.

**Table 4:** Top 15 co-cited references related to stroke motor function rehabilitation.

Rank	Author	Title	Source	Citation	Year	DOI
1	Wolf, SL, et al.	Effect of constraint-induced movement therapy on upper extremity function 3–9 months after stroke – The EXCITE randomized clinical trial	JAMA-J AM MED ASSOC	1,303	2006	10.1177/1545968307305457
2	Murase, N, et al.	Influence of interhemispheric interactions on motor function in chronic stroke	ANN NEUROL	1,014	2004	10.1056/NEJMoa0911341
3	Kwakkel, G, et al.	Effects of robot-assisted therapy on upper limb recovery after stroke: A systematic review	NEUROREHAB NEURAL RE	948	2008	10.1177/1545968307305457
4	Lo, AC, et al.	Robot-Assisted Therapy for Long-Term Upper-Limb Impairment after Stroke	NEW ENGL J MED	856	2010	10.1056/NEJMoa0911341
5	Hummel, F, et al.	Effects of non-invasive cortical stimulation on skilled motor function in chronic stroke	BRAIN	827	2005	10.1093/brain/awh369
6	Laver, KE, et al.	Virtual reality for stroke rehabilitation	COCHRANE DB SYST REV	701	2011	10.1002/14651858.CD008349.pub2
7	Lindvall, O, et al.	Stem cells for the treatment of neurological disorders	NATURE	631	2006	10.1038/nature04960
8	Maciejasz, P, et al.	A survey on robotic devices for upper limb rehabilitation	J NEUROENG REHABIL	618	2014	10.1186/1743-0003-11-3
9	Hummel, FC, et al.	Non-invasive brain stimulation: a new strategy to improve neurorehabilitation after stroke?	LANCET NEUROL	581	2006	10.1016/S1474-4422(06)70525-7
10	Veerbeek, JM, et al.	What Is the Evidence for Physical Therapy Poststroke? A Systematic Review and Meta-Analysis	PLOS ONE	575	2014	10.1371/journal.pone.0087987
11	Clarkson, AN, et al.	Reducing excessive GABA-mediated tonic inhibition promotes functional recovery after stroke	NATURE	570	2010	10.1038/nature09511
12	Ramos-Murguialday, A, et al.	Brain-Machine Interface in Chronic Stroke Rehabilitation: A Controlled Study	ANN NEUROL	543	2013	10.1002/ana.23879
13	Carter, AR, et al.	Resting Interhemispheric Functional Magnetic Resonance Imaging Connectivity Predicts Performance after Stroke	ANN NEUROL	530	2010	10.1002/ana.21905
14	Zhou, HY, et al.	Human motion tracking for rehabilitation-A survey	BIOMED SIGNAL PROCES	472	2008	10.1016/j.bspc.2007.09.001
15	Saposnik, G, et al.	Effectiveness of Virtual Reality Using Wii Gaming Technology in Stroke Rehabilitation A Pilot Randomized Clinical Trial and Proof of Principle	STROKE	464	2010	10.1161/STROKEAHA.110.584979

**Figure 7:**
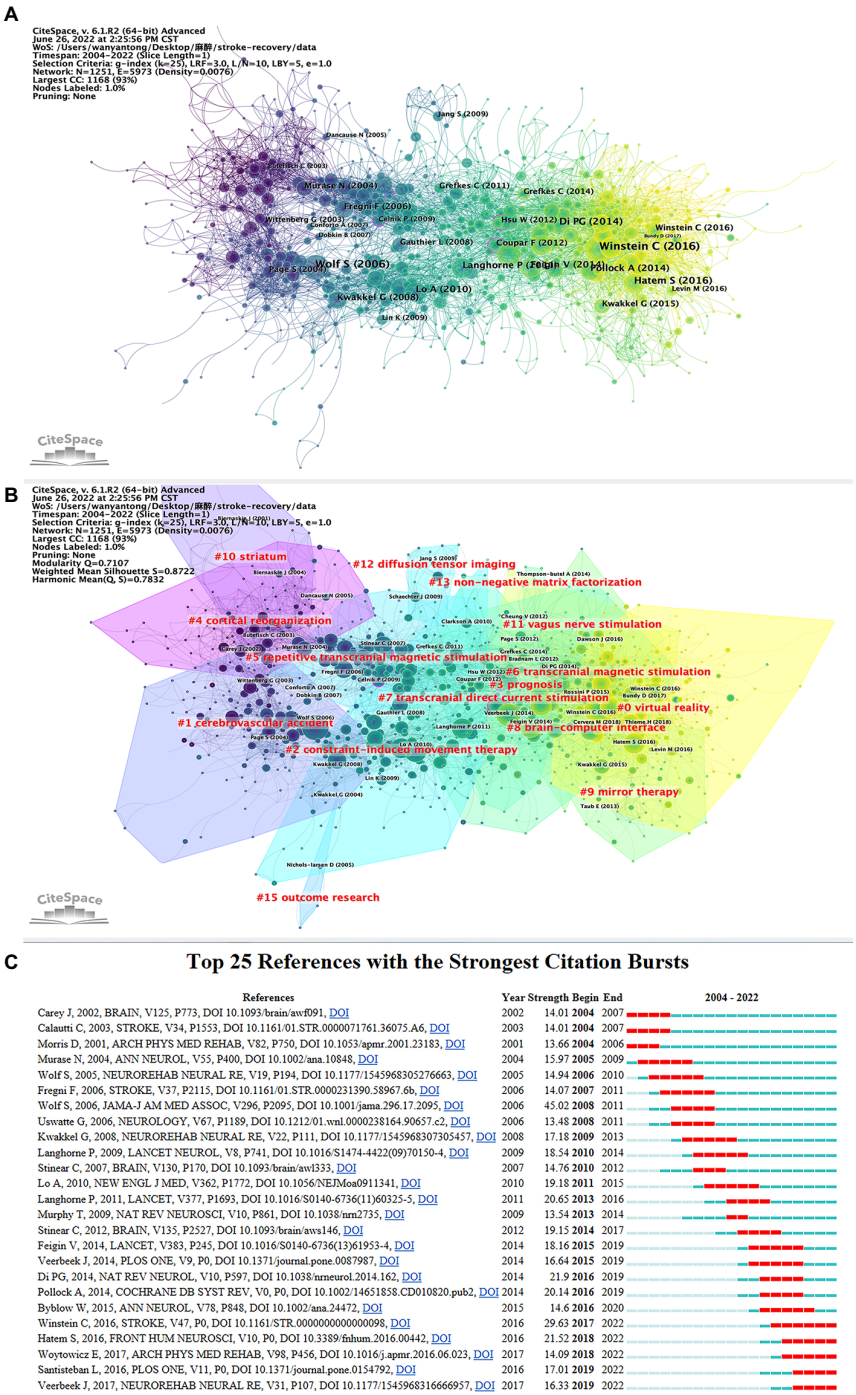
**(A)** Co-cited references related to stroke motor function rehabilitation; **(B)** cluster view of co-cited references in stroke motor function rehabilitation research; **(C)** CiteSpace visualization map of top 25 references with the strongest citation bursts involved in stroke motor function rehabilitation.

Coreference implies that at least two articles are referred to at the same time in at least one report, and the two articles are co-references. Likewise, the bunching capability of CiteSpace to fabricate a visual guide to group the coreference writings was utilized and the recovered written works were partitioned into 15 bunches *via* group examination (Figures 7A,B). Each cluster is closely linked and collaborates in specific areas. The seclusion Q is 0.7107, whereas the typical weighted outline is 0.8722, showing that the bunching structure is steady and has high certainty and persuasiveness. From purple to yellow, Figure 7B depicts the time dimension, which also represents the shift of research focus and direction. The biggest cluster was #0, labeled “virtual,” followed by “cerebrovascular” (cluster #1), “constraint-induced movement therapy” (cluster #2), and “prognosis” (cluster #3). Other important clusters include “cortical reorganization,” “repetitive,” “transcranial magnetic stimulation,” and “transcranial direct current stimulation.”

This study further analyzed the prominent literature. Figure 7C shows the main 25 articles with the most grounded burst force and ranks them in chronological order. In Figure 7C, the research hotspots and time duration can be effectively observed. A large portion of the cocited literature has been referred to in the past 10 years, demonstrating that future examination on stroke may steadily increase.

### Key topics of research hotspots

#### Cluster analysis

**Figure 8:**
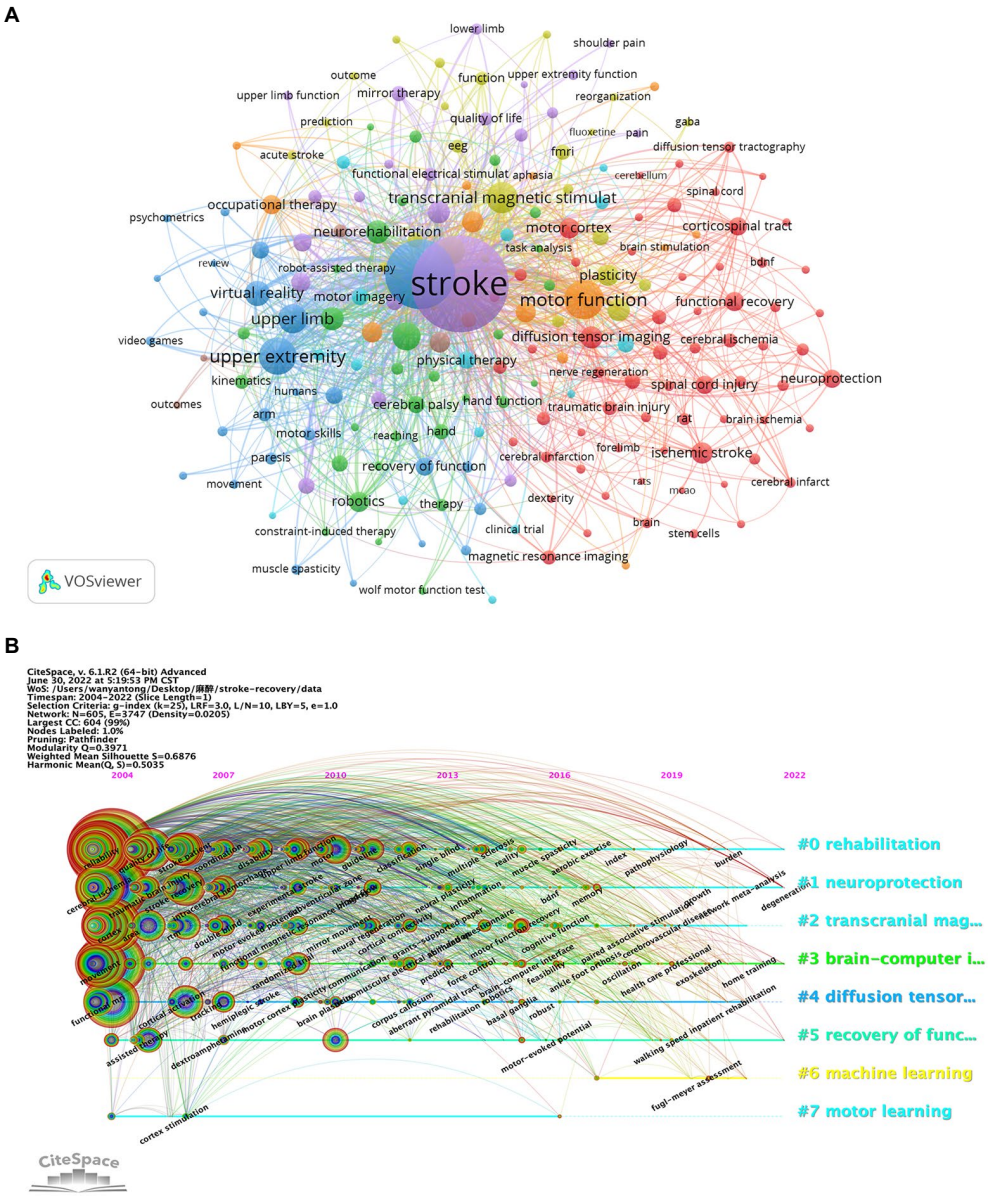
**(A)** Cluster view of keywords in stroke motor function rehabilitation research; **(B)** CiteSpace visualization map of timeline viewer related to stroke motor function rehabilitation research.

Clustering keywords, summarizing research themes, and performing cluster analysis are helpful for understanding the research hotspots. The keywords were grouped using VOSviewer, with circles and labels forming a single unit in the Figure 8A and different colored units representing different clusters. As displayed in Figure 8A, seven clusters are available, each representing seven different search directions. The following are the fundamental catchphrases of the red cluster: diffusion tensor imaging, motor cortex, corticospinal tract, spinal cord injury, and ischemic stroke. The main keywords of the purple cluster include stroke transcranial magnetic stimulus, robot-assisted therapy, and mirror therapy. The keywords of the blue cluster are upper extremity and virtual reality and those of the green cluster are neurorehabilitation and robotics. Other keywords also include motor function and transcranial magnetic stimulus. This indicates the research hotspots and provides some guidance for future researchers.

#### Timeline analysis

Figure 8B depicts a timeline analysis based on VOSviewer mapping software, which intuitively shows the phased research hotspots and development directions of stroke motion recovery from the time dimension. From 2004 to 2012, coordination, traumatic brain injury, cortical activation, assisted therapy, double-blind, randomized trials, guidelines, mirror movement, and other keywords have been widely researched. From 2012 to 2020, research mainly focused on emerging rehabilitation methods of motor function after stroke, and the main keywords used were rehabilitation robotics, brain–computer interface, aerobic exercise, muscle spasm, virtual reality, ankle–foot orthosis, and paired associative stimulation. The interest in studying Fugl-Meyer assessment and exoskeleton began in 2020 and lasted until 2022.

#### Explosion detection

**Figure 9:**
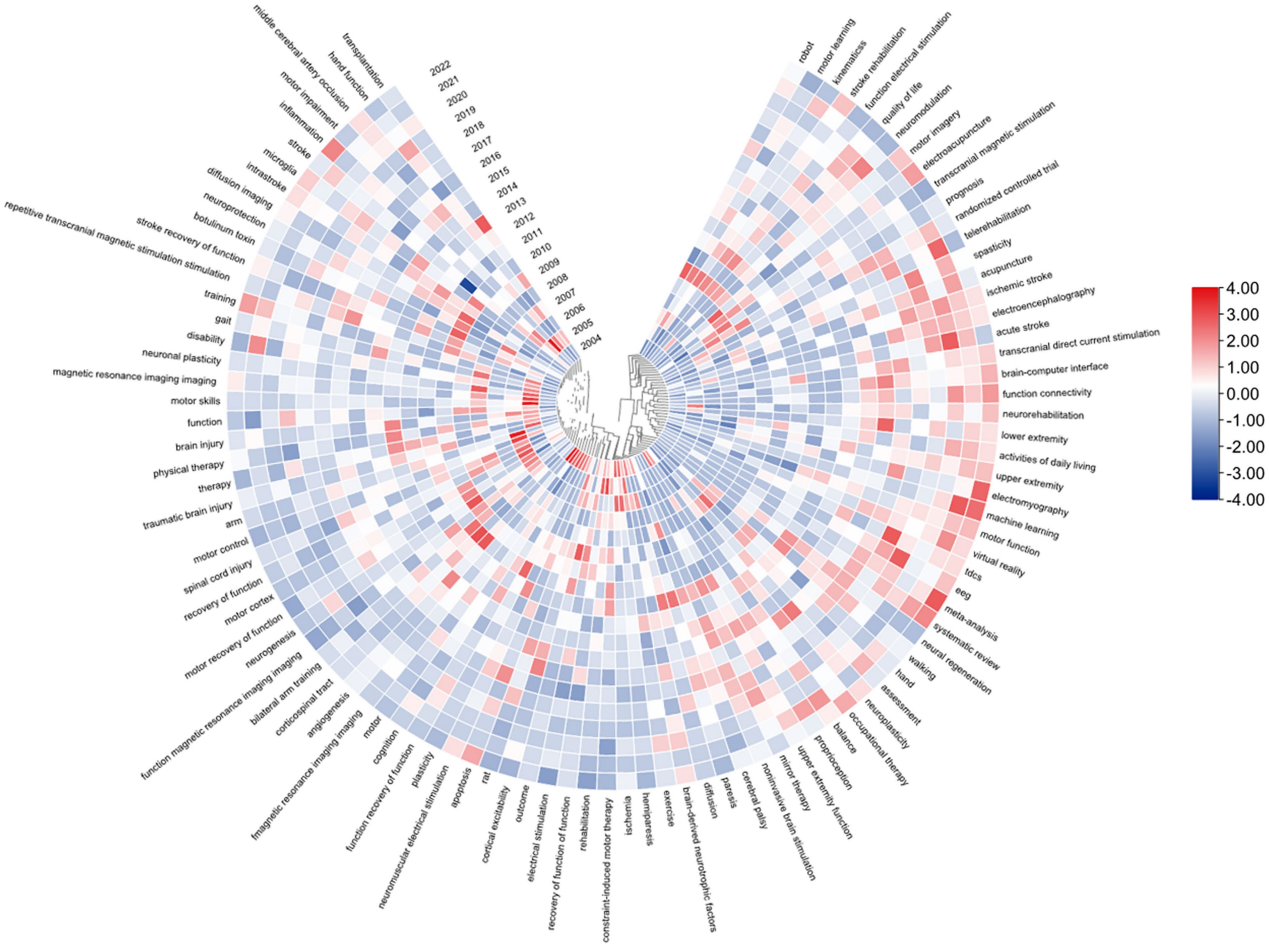
Keywords heat map on stroke motor function recovery.

Catchphrase burst identification alludes to detecting high-frequency keywords for some time, which assists scientists to break down the advancement of examination on the recuperation of motor function after stroke. According to Table 5, excluding “stroke” (1,754), the keywords with high frequency in this study were “rehabilitation” (811), “upper extremity” (390), “motor function” (259), “hemiparesis” (177), and “transcranial magnetic stimulation” (171), suggesting that these fields were the research hotspots. To further visualize the popularity of the keywords, a heat map of the keywords is presented in Figure 9. The value of each grid is divided by the frequency of occurrence by the number of articles distributed annually because as this field shows a rapid growth trend, if only the frequency of occurrence is used, it will produce an error in the number of articles and misjudgment of the popular keywords in that year. As depicted in Figure 8B, changes in future research topics can be intuitively predicted using keywords. Additionally, in Figure 8B, the outer circle denotes the keyword, and the line denotes time. A box replaces the burst intensity of each keyword. The redder the color, more popular the keyword in a particular year. For example, in 2022, the keywords with redder colors were systematic review meta-analysis, machine learning, and electromyography. The popular keywords in recent years are virtual reality, EEG, machine learning, and acute stroke. We can predict that the future research hotspots may be in these areas.

**Table 5:** Top 20 keywords related to stroke motor function rehabilitation.

Rank	Keyword	Occurrences	Total link strength	Rank	Keyword	Occurrences	Total link strength
1	Stroke	1,754	4,560	11	Neurorehabilitation	102	365
2	Rehabilitation	811	2,403	12	Robot	87	360
3	Upper extremity	390	1,258	13	Virtual reality	84	348
4	Motor function	259	723	14	Constraint-induced movement therapy	82	271
5	Hemiparesis	177	547	15	Ischemic stroke	79	167
6	Transcranial magnetic stimulation	171	537	16	Neuroplasticity	73	206
7	Motor recovery	148	408	17	Plasticity	70	208
8	Recovery of function	141	448	18	Diffusion	69	353
9	Stroke rehabilitation	130	337	19	Repetitive transcranial magnetic stimulation	64	253
10	Magnetic resonance imaging	124	361	20	Corticospinal tract	62	217

## Discussion

### Global trends

This study conducted a bibliometric analysis of motor function rehabilitation after stroke from 2004 to 2022. An annual increase in the number of papers and citations published in the journal was observed. The most significant growth period was noted from 2020 to 2021, with the highest number of publications in 2021(Figure 2). Furthermore, research related to motor function rehabilitation after stroke is expected to continue increasing in the future.

In terms of refereed journals, among the top 10 journals, 20%, 50%, 20%, and 10% were Q1, Q2, Q3, and Q4, respectively. *Neurorehabilitation and Neural Repair* was cited the most (9,770 times). One of the top 10 journals had an IF of more than 10 (*Stroke*). Three journals had an IF of 1–3 (*Thomas In Stroke Rehabilitation*, *Restorative Neurology and Neuroscience*, and *Journal of Stroke & Cerebrovascular Diseases*), four journals had an IF of 3–5 (*Neurorehabilitation and Neural Repair*, *Archives of Physical Medicine and Rehabilitation*, *Frontiers in Neurology*, and *PLOS One*), and two journals had an IF of 5–10 (*Journal of Neuroengineering and Rehabilitation* and *Neural Regeneration Research*; Table 3). These results suggest that the quality of research on motor function rehabilitation after stroke is relatively high. The United States (953) was the most significant contributor to the number of papers published in the above mentioned journals, with China (674) and Japan (293) closely behind. Furthermore, the United States had the greatest total link strength (527) and the most citations (45,080). In terms of traditional institutions, the largest cluster was labeled “CI therapy,” followed by “electroacupuncture,” “telemedicine,” and “rehabilitation robotics” (Supplementary Figure S1C). CI therapy, also known as CIMT, is a neurorehabilitation therapy aimed at recuperating the function of an affected limb after stroke. The affected limb is forced to be used instead of the healthy one (Wang et al., 2022). Many studies have confirmed the efficacy of CIMT in improving limb motor function (Ju and Yoon, 2018; Wang et al., 2022). Electroacupuncture therapy combines traditional Chinese medicine acupuncture therapy and Western medicine rehabilitation therapy. When electroacupuncture is applied to both antagonistic and active muscles in patients with spasms after stroke, the intermittent wave improves the excitability of the muscle tissue. The short-term application of low-frequency, continuous waves can also excite the muscles. However, its long-term application can inhibit the sensory and motor nerves (Zhang et al., 2022). Telerehabilitation is developed based on telemedicine owing to the development of telecommunication and information technology (Nikolaev and Nikolaev, 2022). Telecommunication technology, rehabilitation diagnostics, and treatment methods are used to provide rehabilitation services to patients in families, communities, or remote areas (Chumbler et al., 2012). Telerehabilitation is a promising rehabilitation treatment and a realistic choice to deal with coronavirus disease 2019 (COVID-19; Maresca et al., 2020; Suso-Martí et al., 2021). The concept of rehabilitation robotics was developed in engineering. It combines the latest technologies with rehabilitation medicine. Multiple rehabilitation robots are being developed, including upper- and lower-limb rehabilitation robots (Bernhardt and Mehrholz, 2019; Rodgers et al., 2019). Owing to its advantages in improving rehabilitation efficiency, ensuring rehabilitation quality, and reducing human costs, it has gradually become a research hotspot locally and internationally (Morone et al., 2017).

### Research hotspots and frontiers

Articles on motor function rehabilitation after stroke covered several areas, among which rehabilitation, neuroscience, and clinical neurology were the most popular. Other areas included sports sciences, peripheral vascular disease, engineering biomedical, cell biology, medicine, and multidisciplinary sciences, indicating that motor function recovery after stroke is a complex issue warranting multidisciplinary intervention (Figure 5).

One of the most significant factors during the assessment of the quality of research of motor function rehabilitation after stroke is the number of citations. This can help identify the interest areas in the research. After the top 15 frequently cited literature were read and analyzed, the results showed that CIMT, robot-assisted therapy, noninvasive brain stimulation, virtual reality, stem cells, physical therapy, and brain–machine interface were widely concerned by researchers at a particular period in this field (Table 4). The following are the most cited papers that highlight the various research hotspots related to the rehabilitation of patients with stroke. One of these is a multicenter randomized controlled study that reported that rehabilitation using robotic-assisted technology significantly improved the outcomes of patients with long-term extremity dysfunction (Lo et al., 2010). A systematic review of 19 studies involving 565 individuals showed that interactive video games and virtual reality improved activities of daily living functions (Laver et al., 2011). A study involving patients with chronic stroke revealed that training the brain using computer-assisted technology improved motor function. This could form the basis of a new therapeutic approach for treating neurological disorders (Ramos-Murguialday et al., 2013). In the highly cited literature, the main research directions closely correlate to the rehabilitation methods, among which the intelligent rehabilitation technology is the most popular.

A sudden increase in the number of citations of a paper indicates that the paper focuses on a vital issue in the field, known as burst reference. Veerbeek et al. (2017), Santisteban et al. (2016), Woytowicz et al. (2017), Hatem et al. (2016), and Winstein et al. (2016) were the top five strongest burst references in the last 5 years (Figure 7C). A meta-analysis systematically reviewed the impact of robot-assisted treatment on motor function and activities of daily living of the paralyzed arm (Veerbeek et al., 2017). A review showed that Fugl–Meyer assessment was the most commonly used measurement method (Santisteban et al., 2016). A study quantified the upper extremity motor deficits in patients with chronic stroke using cluster analysis of the Fugl–Meyer findings (Woytowicz et al., 2017). A systematic review summarized emerging interventions, including virtual reality, robot-assisted training, and noninvasive brain stimulation (Hatem et al., 2016). This is consistent with most of the previous research hotspots of institutional clustering. The fifth strongest burst reference is the growth-up stroke restoration and recuperation rule (Winstein et al., 2016). In 5 years, except for one guideline, burst references were connected with upper-limb motor function, two with upper-limb motor function assessment, and two with upper-limb motor function rehabilitation methods. Hence, research on the motor function of the upper limb has been popular in the last 5 years. Moreover, this emphasizes the need to reach a worldwide consensus regarding optimal assessment methods of motor function after stroke. The challenge now is to develop evidence-based treatment protocols for different patients.

Clustering analysis of keywords revealed that among the top 10 keywords, alongside the words related to the retrieval words, the study in this field mainly revolved around the upper limbs (top 3), transcranial magnetic stimulation (top 6), and magnetic resonance imaging (top 10; Table 5). Transcranial magnetic stimulation is used for various functional assessments and treatments of the nervous system. It has been proven effective in promoting motor recovery in patients with stroke (Bai et al., 2022). It acts on the central nervous system, changes the membrane potential of cortical neurons, and causes corresponding physiological and biochemical reactions. Magnetic resonance imaging is a standard imaging examination in patients with stroke. Upper limbs and noninvasive brain stimulation are also hot topics in burst literature, indicating that these hotspots will remain popular for some time. Subsequently, combined with timeline analysis and burst detection, an overall understanding of the research progress and future directions of motor function recovery after stroke can be formed, with Fugl–Meyer assessment, electromyography, exoskeleton, home training, telemedicine, and virtual reality becoming the new hot words from 2019 onward (Figures 8B, 9). The main contents are as follows.

#### Motor function evaluation after stroke

The Fugl–Meyer assessment is widely used to assess sensorimotor function after stroke (Santisteban et al., 2016), and electromyography is a popular tool for measuring muscle activity (Cè et al., 2020). Owing to the COVID-19 pandemic, the motor function of several patients with stroke required to be assessed online. Thus, Pérez-Robledo et al. (2022) designed an online Spanish version of the Fugl–Meyer assessment scale. Because the scoring and action guidance of the Fugl–Meyer assessment scale require the participation of professional rehabilitation therapists and the evaluation is difficult to perform at home, Shen and Liu (2022) proposed a video-based Fugl–Meyer assessment system, allowing users to use a camera for at-home Fugl–Meyer assessment. Yamamoto et al. (2020) conducted a cross-sectional study to explore the connection between the results of the upper extremity Fugl–Meyer scale and motor function scores of Functional Independent Measure. Cecchi et al. (2021) developed an Italian version of the Fugl–Meyer assessment by transcultural interpretation and approval. Suponeva et al. (2021) reported high sensitivity, reliability, and validity of the Russian version of the Fugl–Meyer assessment. Rech et al. (2020) concluded that the Fugl–Meyer assessment could be used to assess the quality of motor performance in patients with chronic stroke and different degrees of functional impairment owing to the consistency of the scores as determined with instrumental kinematic measures. Formstone et al. (2021) used a new combination of wearable inertial and mechanical mapping sensors to quantify motor function after stroke, demonstrating that the results of Fugl–Meyer assessment were consistent with those of the mapping sensors. Pan et al. (2021) quantitatively assessed motor function in patients with stroke by collecting surface electromyography (sEMG) signals and inertial sensor data during the voluntary upward reach of the upper extremity. As evidenced by new publications in the last 3 years, the Fugl–Meyer scale has been widely used worldwide. Ongoing research has linguistically and culturally adapted the Fugl–Meyer assessment and confirmed its validity, reliability, and sensitivity. Simultaneously, owing to the outbreak of the COVID-19 pandemic, some studies have proposed online versions of the Fugl–Meyer scale and a video-based Fugl–Meyer assessment system that does not require the involvement of therapists. However, the Fugl–Meyer scale still has some shortcomings, including the imprecision of the scale itself and the variability between different assessors (Formstone et al., 2021). Quantitative assessment of motor function is extremely significant for patients after stroke because it can be used to develop a more precise rehabilitation treatment plan (Pan et al., 2021). Therefore, many automation systems have been proposed for empirical quantification of motor assessments; however, they are insufficient. Clinical practice based on this aspect is required in the future to provide a premise for the broad clinical reception of automated and quantitative instrument applications in poststroke motor assessments.

Electromyography can quantitatively and qualitatively analyze the neuromuscular function of target muscles by collecting surface signals during muscle activity and speculating on the damage extent and nature of target muscles, thereby offering noninvasive and real-time measurements (Cè et al., 2020; Feldner et al., 2021; Fujita et al., 2021; Papazian et al., 2021). Liu et al. (2022) combined sEMG devices and video games to develop a biofeedback-based therapeutic play system, which could improve patient training initiative and compliance. Lee (2022) found that EMG-guided pedaling training was effective for enhancing activities of daily living and improving motor dysfunction in patients with stroke. Khan et al. (2021) proposed a wireless data acquisition system by combining sensors and sEMG, which reduces the dependence of patients on caregivers and enables them to complete daily activities independently by helping them control different devices in real time. Ito et al. (2021) developed an electromyography-controlled gamified movement system that motivates patients with stroke with various degrees of paralysis to complete repetitive finger and wrist movements in enjoyment. Yu et al. (2020) developed an artificial intelligence system using real-time EMG signals that can obtain accurate stroke predictions. This shows that electromyography can potentially be used to predict disease occurrence, and the combination of electromyography and rehabilitation training based on the biofeedback theory is a promising rehabilitation method. Therefore, further development and research are warranted to test the potential efficacy of this combination treatment in the future.

#### Emerging rehabilitation treatment techniques for motor function after stroke

Exoskeleton, home training, telemedicine, and virtual reality are the new keywords from 2020 to 2022. The exoskeleton, a wearable rehabilitation robot, assists patients in daily life activities and gait training and has gained considerable popularity in recent years (Molteni et al., 2017; Nolan et al., 2020). Rodríguez-Fernández et al. (2021) systematically evaluated lower extremity exoskeleton robots to reveal the potential of wearable exoskeletons for applications in early rehabilitation, thereby promoting functional recovery and daily life activities in homes and communities. Chen et al. (2022) developed a tendon-driven soft hand exoskeleton, paving the way for a fully driven soft hand exoskeleton. Chen et al. (2021) proposed a therapeutic system comprising sensory and motor gloves. The sensory gloves worn on the fitness hands contain force and flex sensors, which measured the gripping force and bending angle of each finger joint for motion detection. Simultaneously, micromotors provide an auxiliary driving force for the motor gloves worn on the affected hand to perform training tasks. Exoskeleton equipment has considerably improved, from a large upper and lower limb exoskeleton to a delicate hand exoskeleton and from heavy and bulky devices to comfortable, safe, and soft gloves. However, evidence supporting its benefits has been restricted owing to small study samples, inconsistent clinical protocols, and short intervention durations; therefore, in the future, large-sample, long-term, normalized randomized controlled trials are warranted to demonstrate the clinical effectiveness of the exoskeletons.

Home training is frequently associated with telerehabilitation (Truijen et al., 2022). A review presumed that telerehabilitation is an appropriate option for at-home recovery (Perrochon et al., 2019). Chae et al. (2020) proposed a home rehabilitation system that can be used for at-home treatment of patients with stroke using a smartwatch and cell phone application to identify and record the type and frequency of rehabilitation exercises performed by the user. Cramer et al. (2019) demonstrated that telerehabilitation and general rehabilitation significantly improved arm motor function, indicating that telerehabilitation was as effective as in-hospital rehabilitation. Similarly, Knepley et al. (2021) reviewed articles related to telerehabilitation and concluded that telerehabilitation is less expensive and more effective than in-clinic rehabilitation for improving functional impairment in patients with stroke and that telerehabilitation can be combined with different treatments, including virtual reality. For patients with stroke, the utilization of virtual reality in the rehabilitation process has been proved to improve motor function (Norouzi-Gheidari et al., 2019; Domínguez-Téllez et al., 2020; Karamians et al., 2020; Mekbib et al., 2020). Thielbar et al. (2020) found that multiuser virtual reality training was better for at-home rehabilitation by comparing patient engagement and subjective experience with multiuser and single-user virtual reality therapy. Lee et al. (2020) validated the feasibility of fully immersive virtue reality technology with head mounted displays for the upper-limb recovery of patients with stroke. Qiu et al. (2020) developed a virtual rehabilitation system for home use that can be remotely customized for a certain intensity of upper extremity training. Patients with stroke benefit from longer treatment duration when their motor function improves. However, in the current one-to-one treatment model, increasing the duration of treatment is usually impossible (Norouzi-Gheidari et al., 2019). Therefore, virtual reality and telerehabilitation allow patients to complete the same or even more training at home as in a medical facility (Truijen et al., 2022). Additionally, although there have been more randomized clinical trials performed to test the efficacy of virtual reality, it is difficult to conclude its effectiveness owing to the significant differences between interventions and comparative participants in the different studies. The field is emerging, and stronger examination is expected to have more precise inferences (Laver et al., 2020). Meanwhile, according to Thielbar’s research conclusion (Thielbar et al., 2020), more multiuser-connected rehabilitation systems should be developed based on virtual reality and remote rehabilitation to improve the enthusiasm for rehabilitation training for patients with stroke.

## Limitation

The study had few limitations. First, only the publications distributed in the WoSCC database was incorporated. Second, the selected articles were written in English, which may disregard a few literary works of different language in this field. Therefore, some limitations in literature retrieval occurred. Conversely, recently, the sum of cocitation frequency in the published literature may be rather low owing to the short publishing time, thereby generating contrasts between the study results and genuine circumstances. Finally, because of different algorithms while using CiteSpace to generate visual maps, there are no unitary setting processes of time division, threshold, and clipping methods, which may produce some deviation.

## Conclusion

In this study, we first searched the WoSCC database for the last 18 years of literature regarding poststroke motor function rehabilitation and performed bibliometric and visual analyses using CiteSpace and VOSviewer software, presenting a relatively scientific and intuitive overview of research on this topic. Visual analysis revealed that research in this field is in the stage of rapid development, and the related literature on this topic is constantly emerging, showing a steady growth trend. Briefly, rehabilitation of the upper limb is the most common research hotspot. Regarding rehabilitation evaluation and assessment, the Fugl–Meyer assessment may be the most generally utilized assessment scale. Meanwhile, research on quantitative measurement techniques, including electromyography, has gradually emerged. Regarding rehabilitation treatment technology, in addition to conventional intervention methods, such as CI therapy and noninvasive brain stimulation, research on virtual reality, telemedicine, electroacupuncture, brain–computer interface, and rehabilitation robots, including exoskeleton, has attracted increasing attention. These topics have become research hotspots on poststroke motor function rehabilitation and reveal future research trends. Future research on motor function rehabilitation after stroke should be focused on these points: first, formulate normative and systematic rehabilitation interventions throughout the whole course of stroke, and second, develop targeted interventions for patients with different types and stages of stroke.

## Data availability statement

The original contributions presented in the study are included in the article/Supplementary material, further inquiries can be directed to the corresponding authors.

## Author contributions

DL, JZ, QZ and GH: conception and design, revising the article. JH, JZ, and YW: study conduct and editing the article. YW and PD: data acquisition and analysis. JH, JZ, QY, GL, XW, and LZ: writing the manuscript. All authors contributed to the article and approved the submitted version.

## Funding

This work was supported by the National Natural Science Foundation of China (grant nos. 81874032, 82072528, and 82002380), Natural Science Foundation of Guangdong Province, (2214050003841 to DL, 2022A1515012460 to GH) and College Student’s Innovative Entrepreneurial Training Plan Program (grant no. S202212121168).

## Conflict of interest

The authors declare that the research was conducted in the absence of any commercial or financial relationships that could be construed as a potential conflict of interest.

## Publisher’s note

All claims expressed in this article are solely those of the authors and do not necessarily represent those of their affiliated organizations, or those of the publisher, the editors and the reviewers. Any product that may be evaluated in this article, or claim that may be made by its manufacturer, is not guaranteed or endorsed by the publisher.

## Supplementary material

The Supplementary material for this article can be found online at: https://www.frontiersin.org/articles/10.3389/fnagi.2022.1024163/full#supplementary-material

## References

[ref1] Bai Z. Zhang J. Fong K. N. K. (2022). Effects of transcranial magnetic stimulation in modulating cortical excitability in patients with stroke: a systematic review and meta-analysis. J. Neuroeng. Rehabil. 19:24. doi: 10.1186/s12984-022-00999-4, PMID: 35193624 PMC8862292

[ref2] Bernhardt J. Mehrholz J. (2019). Robotic-assisted training after stroke: RATULS advances science. Lancet 394, 6–8. doi: 10.1016/S0140-6736(19)31156-0, PMID: 31128923

[ref3] Borges L. R. Fernandes A. B. Melo L. P. Guerra R. O. Campos T. F. (2018). Action observation for upper limb rehabilitation after stroke. Cochrane Database Syst. Rev. 2018:CD011887. doi: 10.1002/14651858.CD011887.pub2, PMID: 30380586 PMC6517007

[ref4] Cè E. Longo S. Limonta E. Coratella G. Rampichini S. Esposito F. (2020). Peripheral fatigue: new mechanistic insights from recent technologies. Eur. J. Appl. Physiol. 120, 17–39. doi: 10.1007/s00421-019-04264-w, PMID: 31745629

[ref5] Cecchi F. Carrabba C. Bertolucci F. Castagnoli C. Falsini C. Gnetti B.. (2021). Transcultural translation and validation of Fugl-Meyer assessment to Italian. Disabil. Rehabil. 43, 3717–3722. doi: 10.1080/09638288.2020.1746844, PMID: 32356509

[ref6] Chae S. H. Kim Y. Lee K. S. Park H. S. (2020). Development and clinical evaluation of a web-based upper limb home rehabilitation system using a smartwatch and machine learning model for chronic stroke survivors: prospective comparative study. JMIR Mhealth Uhealth 8:e17216. doi: 10.2196/17216, PMID: 32480361 PMC7380903

[ref7] Chen X. Gong L. Wei L. Yeh S. Da Xu L. Zheng L.. (2021). A wearable hand rehabilitation system with soft gloves. IEEE Trans. Ind. Inf. 17, 943–952. doi: 10.1109/TII.2020.3010369

[ref8] Chen W. Li G. Li N. Wang W. Yu P. Wang R.. (2022). Soft exoskeleton with fully actuated thumb movements for grasping assistance. IEEE Trans. Robotics. 38, 2194–2207. doi: 10.1109/TRO.2022.3148909

[ref9] Chen S. Lu Q. Bai J. Deng C. Wang Y. Zhao Y. (2020). Global publications on stigma between 1998–2018: a bibliometric analysis. J. Affect. Disord. 274, 363–371. doi: 10.1016/j.jad.2020.05.006 32469828

[ref10] Chumbler N. R. Quigley P. Li X. Morey M. Rose D. Sanford J.. (2012). Effects of telerehabilitation on physical function and disability for stroke patients: a randomized, controlled trial. Stroke 43, 2168–2174. doi: 10.1161/STROKEAHA.111.646943, PMID: 22627983

[ref11] Cramer S. C. Dodakian L. Le V. See J. Augsburger R. McKenzie A.. (2019). Efficacy of home-based telerehabilitation vs in-clinic therapy for adults after stroke: a randomized clinical trial. JAMA Neurol. 76, 1079–1087. doi: 10.1001/jamaneurol.2019.1604, PMID: 31233135 PMC6593624

[ref12] Daniel A. Koumans H. Ganti L. (2021). Impact of music therapy on gait after stroke. Cureus 13, –e18441. doi: 10.7759/cureus.18441 PMC8559977 34737909

[ref13] Dimyan M. A. Cohen L. G. (2011). Neuroplasticity in the context of motor rehabilitation after stroke. Nat. Rev. Neurol. 7, 76–85. doi: 10.1038/nrneurol.2010.200, PMID: 21243015 PMC4886719

[ref14] Domínguez-Téllez P. Moral-Muñoz J. A. Salazar A. Casado-Fernández E. Lucena-Antón D. (2020). Game-based virtual reality interventions to improve upper limb motor function and quality of life after stroke: systematic review and meta-analysis. Games Health J. 9, 1–10. doi: 10.1089/g4h.2019.0043, PMID: 32027185

[ref15] Dong X. T. Tan Y. C. Zhuang D. L. Hu T. T. Zhao M. Y. (2022). Global characteristics and trends in research on ferroptosis: a data-driven bibliometric study. Oxidative Med. Cell. Longev. 2022, 1–12. doi: 10.1155/2022/8661864 PMC8787456 35087622

[ref16] Dong Y. L. Weng L. M. Hu Y. H. Mao Y. X. Zhang Y. J. Lu Z. F.. (2022). Exercise for stroke rehabilitation: a bibliometric analysis of global research from 2001 to 2021. Front. Aging Neurosci. 14:876954. doi: 10.3389/fnagi.2022.876954, PMID: 35783146 PMC9247282

[ref17] Efron N. (2021). Exploring the bibliometrics of various ophthalmic fields. Clin. Exp. Optom. 104, 559–560. doi: 10.1080/08164622.2021.1913044, PMID: 34187305

[ref18] Feldner H. A. Papazian C. Peters K. M. Creutzfeldt C. J. Steele K. M. (2021). Clinical use of surface electromyography to track acute upper extremity muscle recovery after stroke: a descriptive case study of a single patient. Appl. Syst. Innov. 4:32. doi: 10.3390/asi4020032 34778722 PMC8589300

[ref19] Feng X. D. Liu C. M. Guo Q. C. Bai Y. J. Ren Y. F. Ren B. B.. (2013). Research progress in rehabilitation treatment of stroke patients a bibliometric analysis. Neural Regen. Res. 8, 1423–1430. doi: 10.3969/j.issn.1673-5374.2013.15.010, PMID: 25206438 PMC4107764

[ref20] Formstone L. Huo W. Wilson S. McGregor A. Bentley P. Vaidyanathan R. (2021). Quantification of motor function post-stroke using novel combination of wearable inertial and mechanomyographic sensors. IEEE Trans. Neural Syst. Rehabil. Eng. 29, 1158–1167. doi: 10.1109/TNSRE.2021.3089613, PMID: 34129501

[ref21] Fujita K. Kobayashi Y. Hitosugi M. (2021). Temporal changes in electromyographic activity and gait ability during extended walking in individuals post-stroke: a pilot study. Healthcare (Basel). 9:444. doi: 10.3390/healthcare9040444 33920156 PMC8070003

[ref22] Hatem S. M. Saussez G. Della Faille M. Prist V. Zhang X. Dispa D.. (2016). Rehabilitation of motor function after stroke: a multiple systematic review focused on techniques to stimulate upper extremity recovery. Front. Hum. Neurosci. 10:442. doi: 10.3389/fnhum.2016.00442 27679565 PMC5020059

[ref23] Isaacs-Itua A. Wong S. C. (2021). Stroke rehabilitation and recovery. Br. J. Hosp. Med. 82, 1–7. doi: 10.12968/hmed.2020.0701 34601931

[ref01] Ito K. Uehara S. Yuasa A. Kim C. M. Kitamura S. Ushizawa K.. (2021). Electromyography-controlled gamified exercise system for the distal upper extremity: a usability assessment in subacute post-stroke patients. Disability and Rehabilitation-Assistive Technology 8, 1–6. 10.1080/17483107.2021.1936663 34102940

[ref24] Ju Y. Yoon I. J. (2018). The effects of modified constraint-induced movement therapy and mirror therapy on upper extremity function and its influence on activities of daily living. J. Phys. Ther. Sci. 30, 77–81. doi: 10.1589/jpts.30.77, PMID: 29410571 PMC5788780

[ref25] Karamians R. Proffitt R. Kline D. Gauthier L. V. (2020). Effectiveness of virtual reality- and gaming-based interventions for upper extremity rehabilitation poststroke: a meta-analysis. Arch. Phys. Med. Rehabil. 101, 885–896. doi: 10.1016/j.apmr.2019.10.195, PMID: 31821799

[ref26] Khan M. A. Bayram B. M. Das R. Puthusserypady S. (2021). Electromyography and inertial motion sensors based wearable data acquisition system for stroke patients: a pilot study. Annu. Int. Conf. IEEE Eng. Med. Biol. Soc. 2021, 6953–6956. doi: 10.1109/EMBC46164.2021.9630245 34892703

[ref27] Knepley K. D. Mao J. Z. Wieczorek P. Okoye F. O. Jain A. P. Harel N. Y. (2021). Impact of telerehabilitation for stroke-related deficits. Telemed. J. E Health 27, 239–246. doi: 10.1089/tmj.2020.0019, PMID: 32326849

[ref28] Kokol P. Blažun Vošner H. Završnik J. (2021). Application of bibliometrics in medicine: a historical bibliometrics analysis. Health Inf. Libr. J. 38, 125–138. doi: 10.1111/hir.12295 31995273

[ref29] Laver K. E. Adey-Wakeling Z. Crotty M. Lannin N. A. George S. Sherrington C. (2020). Telerehabilitation services for stroke. Cochrane Database Syst. Rev. 2020:CD010255. doi: 10.1002/14651858.CD010255.pub3 PMC6992923 32002991

[ref30] Laver K. E. George S. Thomas S. Deutsch J. E. Crotty M. (2011). Virtual reality for stroke rehabilitation. Cochrane Database Syst. Rev.:CD008349. doi: 10.1002/14651858.CD008349.pub2 25927099 PMC6465102

[ref31] Le Danseur M. (2020). Stroke rehabilitation. Crit. Care Nurs. Clin. North Am. 32, 97–108. doi: 10.1016/j.cnc.2019.11.004 32014164

[ref32] Lee K. (2022). EMG-triggered pedaling training on muscle activation, gait, and motor function for stroke patients. Brain Sci. 12:76. doi: 10.3390/brainsci12010076, PMID: 35053819 PMC8773827

[ref33] Lee S. H. Jung H. Y. Yun S. J. Oh B. M. Seo H. G. (2020). Upper extremity rehabilitation using fully immersive virtual reality games with a head mount display: a feasibility study. PM R 12, 257–262. doi: 10.1002/pmrj.12206, PMID: 31218794

[ref34] Liu Y. Silva R. M. L. Friedrich J. B. Kao D. S. Mourad P. D. Bunnell A. E. (2022). Surface electromyography-driven therapeutic gaming for rehabilitation of upper extremity weakness: a pilot study. Plast. Reconstr. Surg. 150, 125–131. doi: 10.1097/PRS.0000000000009208, PMID: 35544314 PMC9246860

[ref35] Lo A. C. Guarino P. D. Richards L. G. Haselkorn J. K. Wittenberg G. F. Federman D. G.. (2010). Robot-assisted therapy for long-term upper-limb impairment after stroke. N. Engl. J. Med. 362, 1772–1783. doi: 10.1056/NEJMoa0911341, PMID: 20400552 PMC5592692

[ref36] Ma D. Yang B. Guan B. Song L. Liu Q. Fan Y.. (2021). A bibliometric analysis of pyroptosis from 2001 to 2021. Front. Immunol. 12:731933. doi: 10.3389/fimmu.2021.731933, PMID: 34484243 PMC8416445

[ref37] Maresca G. Maggio M. G. De Luca R. Manuli A. Tonin P. Pignolo L.. (2020). Tele-neuro-rehabilitation in Italy: state of the art and future perspectives. Front. Neurol. 11:563375. doi: 10.3389/fneur.2020.563375, PMID: 33101176 PMC7554582

[ref38] Mekbib D. B. Han J. Zhang L. Fang S. Jiang H. Zhu J.. (2020). Virtual reality therapy for upper limb rehabilitation in patients with stroke: a meta-analysis of randomized clinical trials. Brain Inj. 34, 456–465. doi: 10.1080/02699052.2020.1725126, PMID: 32064964

[ref39] Molteni F. Gasperini G. Gaffuri M. Colombo M. Giovanzana C. Lorenzon C.. (2017). Wearable robotic exoskeleton for overground gait training in sub-acute and chronic hemiparetic stroke patients: preliminary results. Eur. J. Phys. Rehabil. Med. 53, 676–684. doi: 10.23736/S1973-9087.17.04591-9, PMID: 28118698

[ref40] Morone G. Paolucci S. Cherubini A. De Angelis D. Venturiero V. Coiro P.. (2017). Robot-assisted gait training for stroke patients: current state of the art and perspectives of robotics. Neuropsychiatr. Dis. Treat. 13, 1303–1311. doi: 10.2147/NDT.S114102, PMID: 28553117 PMC5440028

[ref41] Nikolaev V. A. Nikolaev A. A. (2022). Recent trends in telerehabilitation of stroke patients: a narrative review. NeuroRehabilitation 51, 1–22. doi: 10.3233/NRE-210330, PMID: 35527574

[ref42] Nolan K. J. Karunakaran K. K. Chervin K. Monfett M. R. Bapineedu R. K. Jasey N. N.. (2020). Robotic exoskeleton gait training during acute stroke inpatient rehabilitation. Front. Neurorobot. 14:581815. doi: 10.3389/fnbot.2020.581815, PMID: 33192438 PMC7661791

[ref43] Norouzi-Gheidari N. Hernandez A. Archambault P. S. Higgins J. Poissant L. Kairy D. (2019). Feasibility, safety and efficacy of a virtual reality exergame system to supplement upper extremity rehabilitation post-stroke: a pilot randomized clinical trial and proof of principle. Int. J. Environ. Res. Public Health 17:133. doi: 10.3390/ijerph17010113, PMID: 31877910 PMC6981843

[ref44] Osareh F. (1996). Bibliometrics, Citation analysis and Co-Citation analysis: a review of literature I. Libri 46, 149–158. doi: 10.1515/libr.1996.46.3.149

[ref45] Pan B. Huang Z. Jin T. Wu J. Zhang Z. Shen Y. (2021). Motor function assessment of upper limb in stroke patients. J. Healthc. Eng. 2021, 1–11. doi: 10.1155/2021/6621950 PMC7932780 33708365

[ref46] Papazian C. Baicoianu N. A. Peters K. M. Feldner H. A. Steele K. M. (2021). Electromyography recordings detect muscle activity before observable contractions in acute stroke care. Arch. Rehabil. Res. Clin. Transl. 3:100136. doi: 10.1016/j.arrct.2021.100136 34589686 PMC8463445

[ref47] Pérez-Robledo F. Llamas-Ramos R. Llamas-Ramos I. Bermejo-Gil B. M. Sánchez-González J. L. Martín-Nogueras A. M. (2022). Adaptation and feasibility of the online version of the Fugl Meyer scale for the assessment of patients following cerebrovascular accidents. Rev. Neurol. 74, 156–162. doi: 10.33588/rn.7405.2021385, PMID: 35211949 PMC11502211

[ref48] Perrochon A. Borel B. Istrate D. Compagnat M. Daviet J. C. (2019). Exercise-based games interventions at home in individuals with a neurological disease: a systematic review and meta-analysis. Ann. Phys. Rehabil. Med. 62, 366–378. doi: 10.1016/j.rehab.2019.04.004, PMID: 31078706

[ref49] Pitt K. M. McCarthy J. W. (2021). Strategies for highlighting items within visual scene displays to support augmentative and alternative communication access for those with physical impairments. Disabil. Rehabil. Assist. Technol., 1–6. 10.1080/17483107.2021.2003455 34788177

[ref50] Qiu Q. Cronce A. Patel J. Fluet G. G. Mont A. J. Merians A. S.. (2020). Development of the home based virtual rehabilitation system (HoVRS) to remotely deliver an intense and customized upper extremity training. J. Neuroeng. Rehabil. 17:155. doi: 10.1186/s12984-020-00789-w 33228709 PMC7685660

[ref51] Ramos-Murguialday A. Broetz D. Rea M. Läer L. Yilmaz O. Brasil F. L.. (2013). Brain-machine interface in chronic stroke rehabilitation: a controlled study. Ann. Neurol. 74, 100–108. doi: 10.1002/ana.23879, PMID: 23494615 PMC3700597

[ref52] Rech K. D. Salazar A. P. Marchese R. R. Schifino G. Cimolin V. Pagnussat A. S. (2020). Fugl-Meyer assessment scores are related with kinematic measures in people with chronic hemiparesis after stroke. J. Stroke Cerebrovasc. Dis. 29:104463. doi: 10.1016/j.jstrokecerebrovasdis.2019.104463, PMID: 31740027

[ref53] Rodgers H. Bosomworth H. Krebs H. I. van Wijck F. Howel D. Wilson N.. (2019). Robot assisted training for the upper limb after stroke (RATULS): a multicentre randomised controlled trial. Lancet 394, 51–62. doi: 10.1016/S0140-6736(19)31055-4, PMID: 31128926 PMC6620612

[ref54] Rodríguez-Fernández A. Lobo-Prat J. Font-Llagunes J. M. (2021). Systematic review on wearable lower-limb exoskeletons for gait training in neuromuscular impairments. J. Neuroeng. Rehabil. 18:22. doi: 10.1186/s12984-021-00815-5, PMID: 33526065 PMC7852187

[ref55] Saavedra-García A. Moral-Munoz J. A. Lucena-Anton D. (2021). Mirror therapy simultaneously combined with electrical stimulation for upper limb motor function recovery after stroke: a systematic review and meta-analysis of randomized controlled trials. Clin. Rehabil. 35, 39–50. doi: 10.1177/0269215520951935, PMID: 32830512

[ref56] Santisteban L. Térémetz M. Bleton J. P. Baron J. C. Maier M. A. Lindberg P. G. (2016). Upper limb outcome measures used in stroke rehabilitation studies: a systematic literature review. PLoS One 11:e0154792. doi: 10.1371/journal.pone.0154792, PMID: 27152853 PMC4859525

[ref57] Shen X. W. Liu X. (2022). Automatic Fugl-Meyer assessment based on videos. J. Electron. Meas. Instrum. 36, 1–11. doi: 10.13382/j.jemi.B2104225

[ref58] Stinear C. M. Lang C. E. Zeiler S. Byblow W. D. (2020). Advances and challenges in stroke rehabilitation. Lancet Neurol. 19, 348–360. doi: 10.1016/S1474-4422(19)30415-6, PMID: 32004440

[ref59] Suponeva N. A. Yusupova D. G. Zimin A. A. Rimkevichus A. A. Melchenko D. A. Ilyina K. A.. (2021). Validation of the Russian version of the Fugl-Meyer assessment of physical performance for assessment of patients with post-stroke paresis. Zh. Nevrol. Psikhiatr. Im. S S Korsakova 121, 86–90. doi: 10.17116/jnevro202112108286, PMID: 34553587

[ref60] Suso-Martí L. La Touche R. Herranz-Gómez A. Angulo-Díaz-Parreño S. Paris-Alemany A. Cuenca-Martínez F. (2021). Effectiveness of telerehabilitation in physical therapist practice: an umbrella and mapping review with meta–meta-analysis. Phys. Ther. 101:pzab075. doi: 10.1093/ptj/pzab075, PMID: 33611598 PMC7928612

[ref61] Synnestvedt M. B. Chen C. Holmes J. H. (2005). CiteSpace II: visualization and knowledge discovery in bibliographic databases. AMIA Annu. Symp. Proc. 2005, 724–728. PMID: 16779135 PMC1560567

[ref62] Thielbar K. O. Triandafilou K. M. Barry A. J. Yuan N. Nishimoto A. Johnson J.. (2020). Home-based upper extremity stroke therapy using a multiuser virtual reality environment: a randomized trial. Arch. Phys. Med. Rehabil. 101, 196–203. doi: 10.1016/j.apmr.2019.10.182, PMID: 31715140

[ref63] Timmermans C. Roerdink M. Meskers C. G. M. Beek P. J. Janssen T. W. J. (2021). Walking-adaptability therapy after stroke: results of a randomized controlled trial. Trials 22:923. doi: 10.1186/s13063-021-05742-3, PMID: 34911566 PMC8672482

[ref64] Truijen S. Abdullahi A. Bijsterbosch D. van Zoest E. Conijn M. Wang Y.. (2022). Effect of home-based virtual reality training and telerehabilitation on balance in individuals with Parkinson disease, multiple sclerosis, and stroke: a systematic review and meta-analysis. Neurol. Sci. 43, 2995–3006. doi: 10.1007/s10072-021-05855-2, PMID: 35175439 PMC9023738

[ref65] Ugolini D. Neri M. Cesario A. Marazzi G. Milazzo D. Volterrani M.. (2013). Bibliometric analysis of literature in cerebrovascular and cardiovascular diseases rehabilitation: growing numbers, reducing impact factor. Arch. Phys. Med. Rehabil. 94, 324–331.e1. doi: 10.1016/j.apmr.2012.08.205, PMID: 22922328

[ref66] Veerbeek J. M. Langbroek-Amersfoort A. C. van Wegen E. E. Meskers C. G. Kwakkel G. (2017). Effects of robot-assisted therapy for the upper limb after stroke: a systematic review and meta-analysis. Neurorehabil. Neural Repair 31, 107–121. doi: 10.1177/1545968316666957 27597165

[ref67] Wang D. Xiang J. He Y. Yuan M. Dong L. Ye Z.. (2022). The mechanism and clinical application of constraint-induced movement therapy in stroke rehabilitation. Front. Behav. Neurosci. 16:828599. doi: 10.3389/fnbeh.2022.828599, PMID: 35801093 PMC9253547

[ref68] Winstein C. J. Stein J. Arena R. Bates B. Cherney L. R. Cramer S. C.. (2016). Guidelines for adult stroke rehabilitation and recovery: a guideline for healthcare professionals from the American Heart Association/American Stroke Association. Stroke 47, e98–e169. doi: 10.1161/STR.0000000000000098, PMID: 27145936

[ref69] Woytowicz E. J. Rietschel J. C. Goodman R. N. Conroy S. S. Sorkin J. D. Whitall J.. (2017). Determining levels of upper extremity movement impairment by applying a cluster analysis to the Fugl-Meyer assessment of the upper extremity in chronic stroke. Arch. Phys. Med. Rehabil. 98, 456–462. doi: 10.1016/j.apmr.2016.06.023, PMID: 27519928 PMC5299057

[ref70] Yamamoto H. Takeda K. Koyama S. Morishima K. Hirakawa Y. Motoya I.. (2020). Relationship between upper limb motor function and activities of daily living after removing the influence of lower limb motor function in subacute patients with stroke: a cross-sectional study. Hong Kong J. Occup. Ther. 33, 12–17. doi: 10.1177/156w.9186120926609, PMID: 33815019 PMC8008369

[ref71] Yao R. Q. Ren C. Wang J. N. Wu G. S. Zhu X. M. Xia Z. F.. (2020). Publication trends of research on sepsis and host immune response during 1999–2019: a 20-year bibliometric analysis. Int. J. Biol. Sci. 16, 27–37. doi: 10.7150/ijbs.37496, PMID: 31892843 PMC6930382

[ref72] Yu J. Kwon S.-H. Ho C. M. B. Pyo C.-S. Lee H. (2020). Stroke disease prediction system using real-time electromyography signals. Appl. Sci.-Basel. 10:6791. doi: 10.3390/app10196791

[ref73] Zhang J. B. Wei R. P. Yang H. Han B. (2022). Effect of electroacupuncture at antagonistic muscle and agonistic muscle on motor function in patients with upper-extremity spasticity after stroke. Zhongguo zhen jiu = Chinese acupuncture & moxibustion. 42, 381–384. doi: 10.13703/j.0255-2930.20210406-k0001, PMID: 35403395

[ref74] Zhou M. Wang R. Cheng S. Xu Y. Luo S. Zhang Y.. (2021). Bibliometrics and visualization analysis regarding research on the development of microplastics. Environ. Sci. Pollut. Res. 28, 8953–8967. doi: 10.1007/s11356-021-12366-2, PMID: 33447976

