# Supplementary figures and images for: Rehabilitation of motor function after stroke: A bibliometric analysis of global research from 2004 to 2022
Source: Front Aging Neurosci. 2022 Nov 2;14:1024163. doi: 10.3389/fnagi.2022.1024163 (PMC9667945; doi:10.3389/fnagi.2022.1024163)

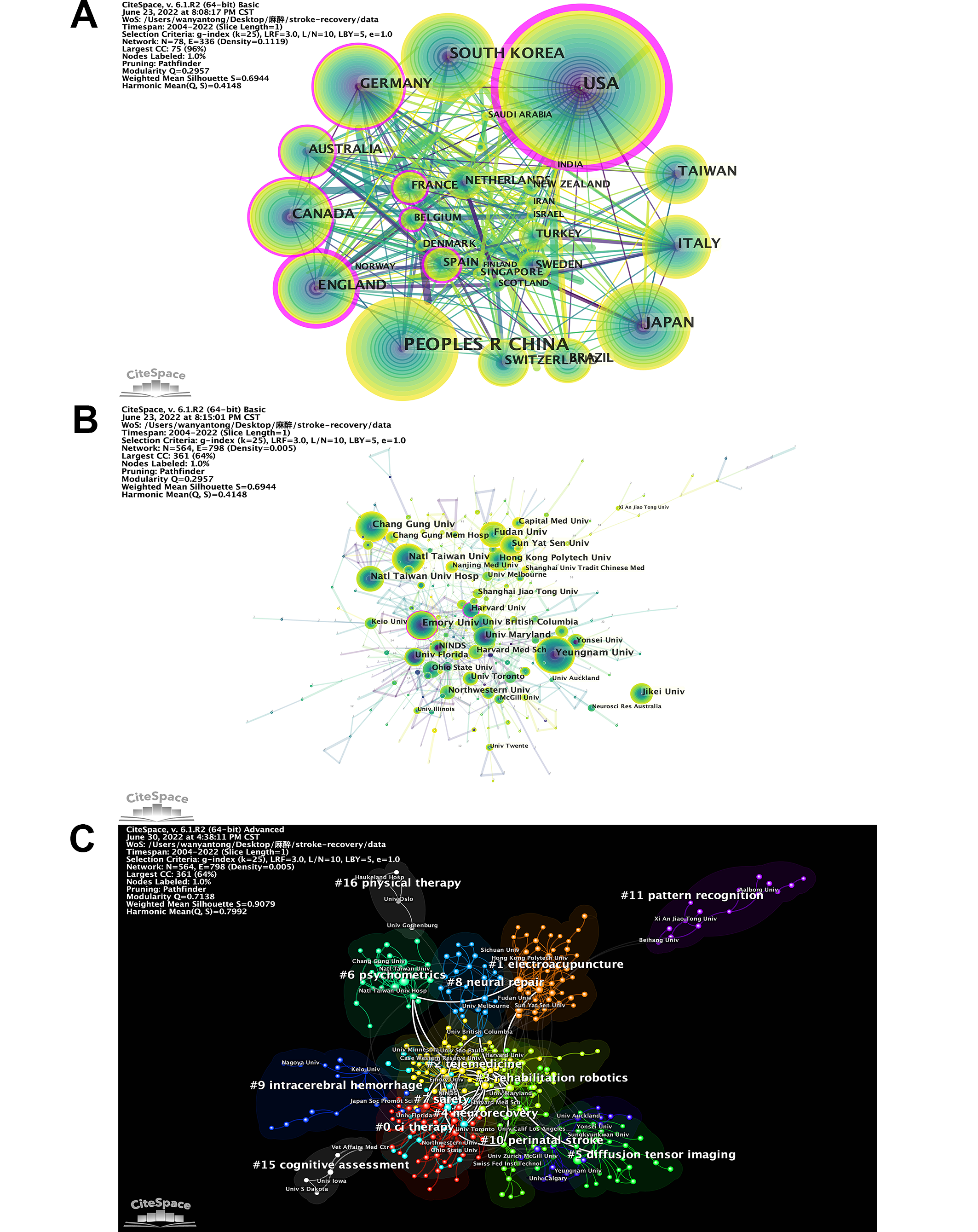

Supplement: Supplementary file 2 [file Image_1.TIF]

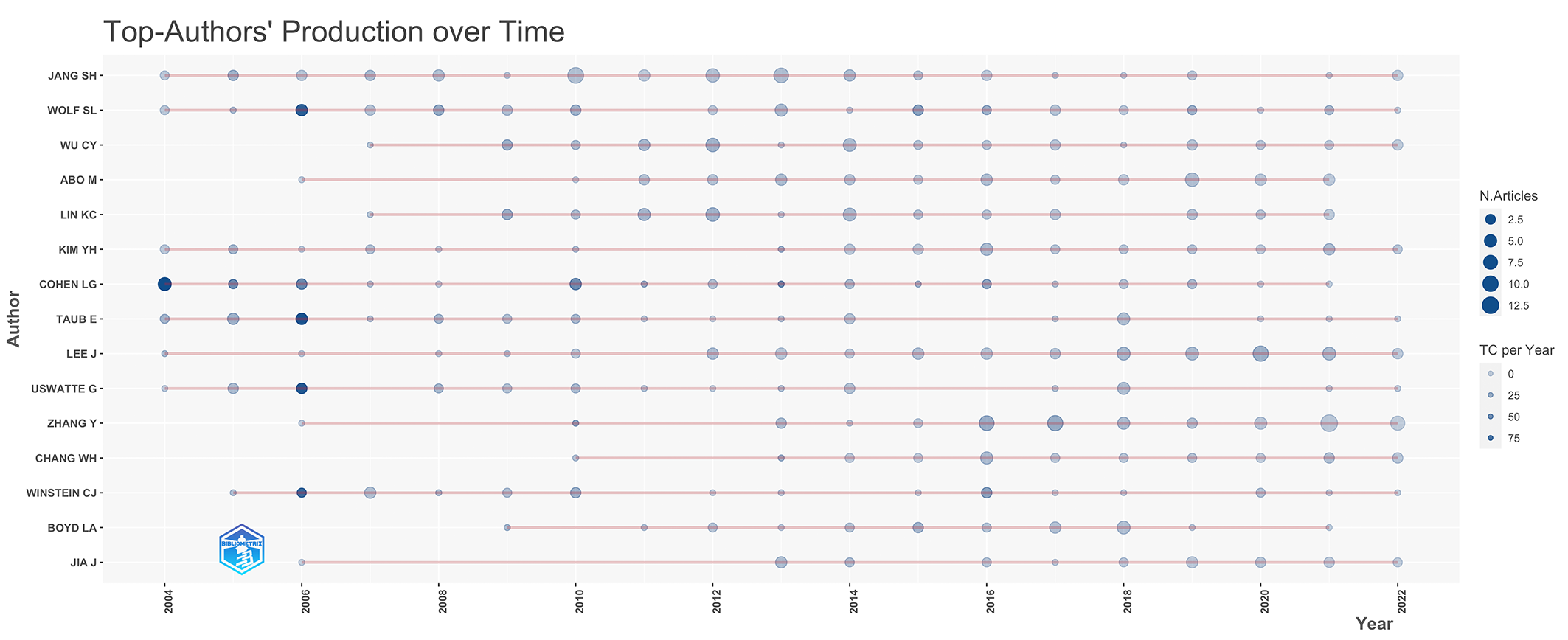

Supplement: Supplementary file 3 [file Image_2.TIF]
